# Analysis of Co-inhibitory Receptor Expression in COVID-19 Infection Compared to Acute *Plasmodium falciparum* Malaria: LAG-3 and TIM-3 Correlate With T Cell Activation and Course of Disease

**DOI:** 10.3389/fimmu.2020.01870

**Published:** 2020-08-26

**Authors:** Marissa Herrmann, Sophia Schulte, Nils H. Wildner, Melanie Wittner, Thomas Theo Brehm, Michael Ramharter, Robin Woost, Ansgar W. Lohse, Thomas Jacobs, Julian Schulze zur Wiesch

**Affiliations:** ^1^Infectious Diseases Unit, I. Department of Medicine, University Medical Center Hamburg-Eppendorf, Hamburg, Germany; ^2^German Center for Infection Research (DZIF), Partner Site Hamburg-Lübeck-Borstel-Riems, Hamburg, Germany; ^3^Department of Tropical Medicine, Bernhard-Nocht-Institute for Tropical Medicine (BNITM), Hamburg, Germany; ^4^Protozoa Immunology, Bernhard-Nocht-Institute for Tropical Medicine, Hamburg, Germany

**Keywords:** COVID-19, malaria, SARS-CoV-2, *Plasmodium falciparum*, T cells, PD-1, LAG-3, TIM-3

## Abstract

Coronavirus disease 2019 (COVID-19) which is caused by the novel SARS-CoV-2 virus is a severe flu-like illness which is associated with hyperinflammation and immune dysfunction. The virus induces a strong T and B cell response but little is known about the immune pathology of this viral infection. Acute *Plasmodium falciparum* malaria also causes acute clinical illness and is characterized by hyperinflammation due to the strong production of pro-inflammatory cytokines and a massive activation of T cells. In malaria, T cells express a variety of co-inhibitory receptors which might be a consequence of their activation but also might limit their overwhelming function. Thus, T cells are implicated in protection as well as in pathology. The outcome of malaria is thought to be a consequence of the balance between co-activation and co-inhibition of T cells. Following the hypothesis that T cells in COVID-19 might have a similar, dual function, we comprehensively characterized the differentiation (CCR7, CD45RO) and activation status (HLA-DR, CD38, CD69, CD226), the co-expression of co-inhibitory molecules (PD1, TIM-3, LAG-3, BTLA, TIGIT), as well as the expression pattern of the transcription factors T-bet and eomes of CD8^+^ and CD4^+^ T cells of PBMC of *n* = 20 SARS-CoV-2 patients compared to *n* = 10 *P. falciparum* infected patients and *n* = 13 healthy controls. Overall, acute COVID-19 and malaria infection resulted in a comparably elevated activation and altered differentiation status of the CD8^+^ and CD4^+^ T cell populations. T effector cells of COVID-19 and malaria patients showed higher frequencies of the inhibitory receptors T-cell immunoglobulin mucin-3 (TIM-3) and Lymphocyte-activation gene-3 (LAG-3) which was linked to increased activation levels and an upregulation of the transcription factors T-bet and eomes. COVID-19 patients with a more severe disease course showed higher levels of LAG-3 and TIM-3 than patients with a mild disease course. During recovery, a rapid normalization of these inhibitory receptors could be observed. In summary, comparing the expression of different co-inhibitory molecules in CD8^+^ and CD4^+^ T cells in COVID-19 vs. malaria, there is a transient increase of the expression of certain inhibitory receptors like LAG-3 and TIM-3 in COVID-19 in the overall context of acute immune activation.

## Introduction

Since December 2019, when the emerging Coronavirus SARS-CoV-2 was first described in Wuhan, China, a rapid increase in cases and deaths worldwide could be recorded. The virus, responsible for Coronavirus disease 2019 (COVID-19), belongs to the coronavirus family and causes respiratory tract infections. SARS-CoV-2 is a 29.903 nucleotide large, single-strand RNA virus coding for four structural proteins: the spike protein, the membrane protein, the envelope protein, and the nucleocapsid protein (1, 2). Previous studies have shown that recovered COVID-19 patients developed anti-spike-protein immunity (3).

While most patients are asymptomatic or display only mild symptoms such as fatigue, fever, and dry coughs, some individuals develop pneumonia, severe acute respiratory distress syndrome, sepsis, and septic shock with an overall case fatality rate of 5% (4–6). In a subset of patients, COVID-19 is associated with a so-called cytokine storm, lymphopenia, and dysregulation of the immune system, yet the underlying mechanisms and cellular sources of these immunological complications remain to be fully understood (7, 8). First studies have shown that reduced T cell counts correlated with disease severity in COVID-19 patients (9). Broadly directed, antigen-specific T cell responses could be detected in the effector and central memory subsets of CD8^+^ and CD4^+^ T cells of COVID-19 patients (1, 10). CD8^+^ T cells predominantly produced IFN-γ, while CD4^+^ T cells produced Th1 and Th2 cytokines (1, 10). The exact role and function of T cells in COVID-19 patients with regards to immunological complications like cytokine storm, however, needs to be elucidated in further studies (11).

From other viral infections, we know that cytotoxic T cells play an important role in killing virus-infected cells and thus contribute to the clearance of the infection (12, 13). Detailed studies of the phenotype and function of virus-specific T cells are important to understand their potential role in the pathophysiology of the disease and to develop future treatment strategies (14–16). In order to induce effector T cells with high proliferative and cytotoxic activity and to prevent excessive host immune responses, a balanced T cell response is shaped by the simultaneous upregulation of co-stimulatory and co-inhibitory receptors (17, 18). Inhibitory receptors and their role in T cell exhaustion have been extensively studied in cancer as well as chronic infections such as HCV and HIV (14, 19–23). However, in the early stages of an acute infection, the kinetics and role of co-inhibitory molecules are not well-understood. It is not clear how early T cell exhaustion and loss of effector function set in and whether exhaustion plays a role in the pathophysiology of acute infections (9, 17, 24, 25). Previous studies of T cells and their role in COVID-19 patients have shown an upregulation of inhibitory receptors like PD1. Therefore, it has been suggested that T cell exhaustion might play a role in the pathophysiology of COVID-19 infection (9). However, an upregulation of inhibitory receptors in acute infections does not necessarily correlate with terminal exhaustion of these cells but can be regarded rather as a characteristic of the overall immune activation to counterbalance excessive immune responses (17). A similar, strong up-regulation of a combination of several co-inhibitory receptors in the context of massive T cell activation and secretion of pro-inflammatory cytokines has been observed in acute malaria (26–28). It has been shown that the expression of these co-inhibitory receptors has a double-edged role with potentially detrimental, but also beneficial effects (26, 29–32): on the one hand, over-expression of inhibitory receptors can hinder the hosts ability to clear the infection. On the other hand, this inhibition of immune cells can also dampen hyperinflammation by down-regulating T cell effector functions (29, 31). These studies highlight the importance of intricate modulation of the T cell response in malaria rather than supporting the concept of development of terminal T cell exhaustion at early stages of infection. We hypothesize that T cells are modulated in a similar way in COVID-19 and thus compared the pattern of co-inhibitory receptor expression in malaria vs. COVID-19. In the case of immunological analysis of patients with COVID-19, it will be important to assess the level of co-expression and the ranges of the different co-inhibitory receptors to understand their adverse effects and interpret their role in the overall context of acute infection (33, 34). Comparing the T cell response in COVID-19 patients not only with samples of healthy individuals but also with samples of patients with other acute infections will help to identify the specific immune signature of COVID-19 infection.

In this current study we comprehensively examined the T cell expression profile of inhibitory and stimulatory receptors as well as the expression of the transcription factors T-bet and eomes in a cohort of *n* = 20 COVID-19 and *n* = 10 malaria patients in order to obtain a more detailed understanding of the nature of T cells in the context of acute infection and disease severity. Both, acute infection with SARS-CoV-2 and *Plasmodium falciparum*, can cause excessive host immune activation that can in terms cause severe damage and even limit the hosts ability to clear the disease (8, 28).

## Materials and Methods

### Patient Cohort

PBMC (peripheral blood mononuclear cells) of SARS-CoV-2 infected patients (*n* = 20) and *P. falciparum* infected patients (*n* = 10) as well as uninfected healthy individuals (*n* = 13) were collected at the University Medical Center Hamburg Eppendorf. Blood samples of *P. falciparum* infected patients and healthy controls were collected prior to the COVID-19 outbreak. The study was approved by the local ethics board of the Ärztekammer Hamburg (PV4238, PV4780, PV7298) and written consent was obtained by all study participants. SARS-CoV-2 infection was verified by RT-PCR of nasopharyngeal swabs as previously described (35). *Plasmodium falciparum* infection was confirmed microscopically at the Bernhard-Nocht-Institute for Tropical Medicine. Thick and thin blood smears were stained with 4% Giemsa and examined under oil immersion (original magnification ×100). Clinical and laboratory data were obtained by structured chart review.

### Intracellular Staining and Flow Cytometry

Intracellular as well as surface staining was performed as previously described (36). Cryopreserved PBMC were thawed and stained with the LIVE/DEAD™ Fixable Near-IR dye (Thermo Fisher, Schwerte, Germany). Cells were then stained with the following surface antibodies: anti-CD3 (clone UCHT1, Biolegend), anti-CD8 (clone RPA-T8, Biolegend), anti-CD4 (clone SK3, BD Biosciences), anti-CD19 (clone HIB19, Biolegend), anti-CD14 (clone 63D3, Biolegend), anti-CD45RO (clone UCHL1, Biolegend), anti-CCR7 (clone G043H7, Biolegend), anti-CD27 (clone M-T271, BD Biosciences), anti-CD127 (clone A019D5, Biolegend) anti-CD69 (clone FN50, Biolegend), anti-CD38 (clone HB7, BD Biosciences), anti-HLA-DR (clone L243, Biolegend), anti-CD226 (clone DX11, BD Biosciences), anti-PD1 (clone EH12.2H7, Biolegend), anti-LAG-3 (clone 3D-S223H or 11C3C65, eBioscience or Biolegend), anti-TIM-3 (clone F38-2E2, Biolegend), anti-BTLA (clone J168-540, BD Biosciences), and anti-TIGIT (clone A15153, Biolegend) for 20 min at room temperature in the dark. After fixation and permeabilization using the eBioscienceTM Foxp3/Transcription Factor Staining Buffer Set cells were incubated with anti-T-bet (clone 4B10, Biolegend) and anti-eomes (clone WD1928, Invitrogen) for 30 min at room temperature in the dark. The cells were analyzed on a BD LSRFortessa using the FACS Diva version 8 (BD Biosciences). An overview of the fluorochrome-conjugated antibodies and processed samples is given in Supplementary Figures 1, 2.

### Statistical Analysis

Flow cytometric data was analyzed with FlowJo version 10 (Treestar, Ashland, OR, USA). Statistical analysis was performed using the GraphPad Prism 8 software (GraphPad Software, San Diego, CA). Unpaired groups were analyzed using the Mann–Whitney test while paired groups were analyzed with the Wilcoxon test. For bivariate correlation analysis the Spearman correlation was applied. Data are expressed as mean with standard deviation. *P* < 0.05 were considered to be significant.

## Results

### Clinical Characteristics of the COVID-19 and Malaria Cohort

**Figure 1:**
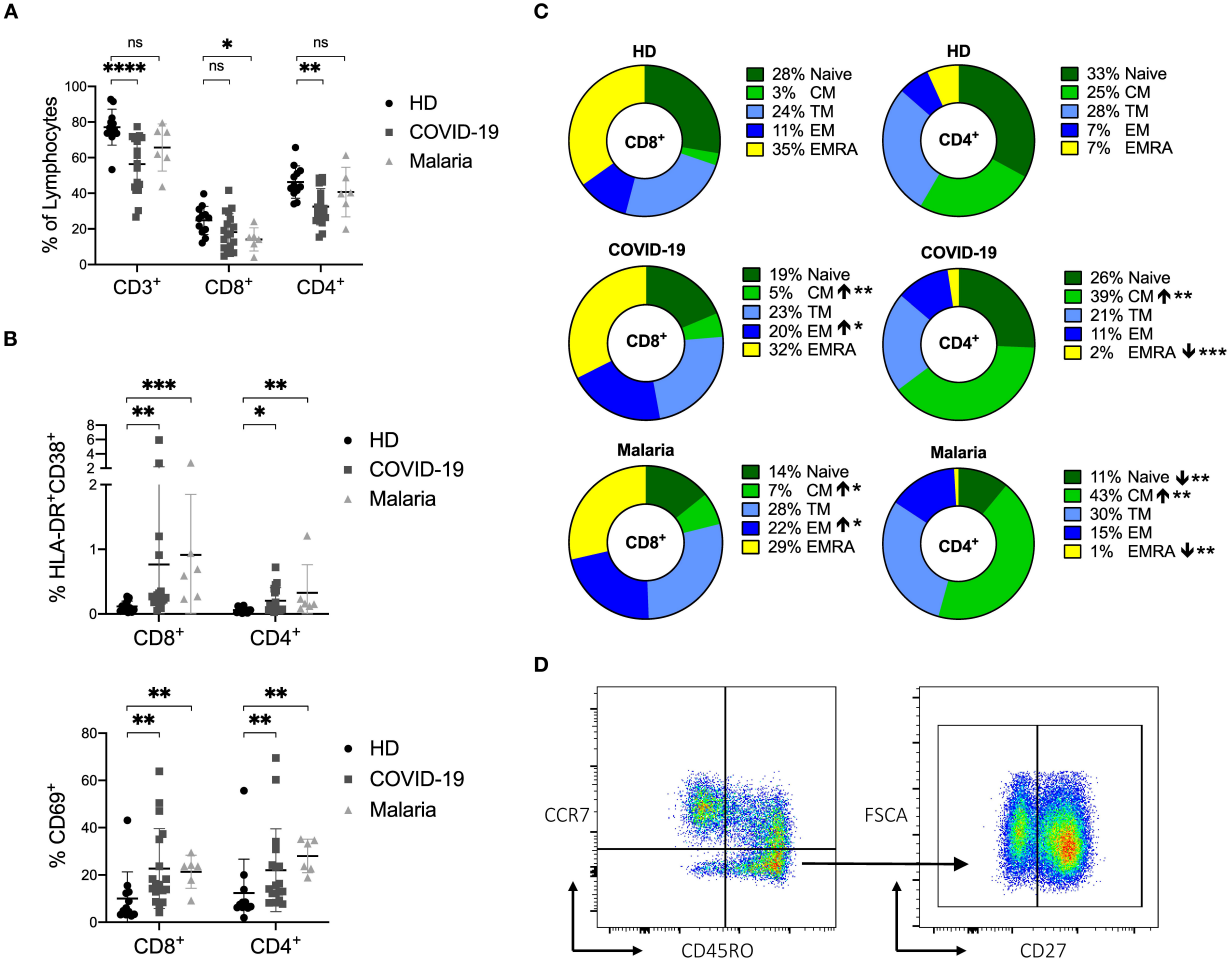
**(A–D)** Frequency of CD3^+^ T cells and distribution of naïve and memory subsets as well as the activation status of T cells in COVID-19 and malaria patients compared to healthy donors. **(A)** Frequency of CD3^+^, CD8^+^, and CD4^+^ T cells in healthy donors (HD) and COVID-19 and malaria patients. **(B)** Frequency of CD69^+^, HLA-DR^+^CD38^+^ CD8^+^, and CD4^+^ T cells in healthy donors and COVID-19 and malaria patients. **(C)** T cell distribution illustrated in donut charts showing the mean frequency of naive (CCR7^+^/CD45RO^−^), central memory CM (CCR7^+^/CD45RO^−^), transitional memory TM (CCR7^−^/CD45RO^+^/CD27^+^), effector memory EM (CCR7^−^/CD45RO^+^/CD27^−^), and terminal effector memory EMRA (CCR7^−^/CD45RO^−^) CD8^+^, and CD4^+^ T cells in healthy donors and COVID-19 and malaria patients. Frequencies in COVID-19 and malaria patients were compared with HD. **(D)** Representative dot plots of PBMC gated on either CD8^+^ or CD4^+^ T cells for T cell distribution. *P*-values were calculated by Mann–Whitney test. *P*-values smaller than 0.05 were considered significant, where *, **, ***, and **** indicate *p*-values between 0.01 to 0.05, 0.001 to 0.01, 0.0001 to 0.001 and <0.0001 respectively.

The clinical cohorts consisted of 20 SARS-CoV-2 and 10 *P. falciparum* infected patients and 13 healthy individuals. All patients were admitted to the University Medical Center Hamburg Eppendorf due to the severity of their symptoms. None of the patients needed to be admitted to the intensive care unit at the time of blood collection. During the course of the hospital stay, a total of three COVID-19 patients were transferred to the intensive care unit, one of whom unfortunately later died due to a pulmonary embolism. On average, the COVID-19 patients spent 9.9 days and the malaria patients spent 4.9 days in the hospital. The mean age of the COVID-19 patients was 54 years compared to 55 years of patients with malaria. Of note, one COVID-19 patient had received treatment with Rituximab 3 months prior to blood sample collection and another patient had received treatment with a PD-1 antibody several weeks prior to the infection with SARS-CoV-2. We enrolled PBMC of 13 healthy volunteers into our study, eight female and five male with a mean age of 35 ± 8. The analysis of lymphocyte subsets in the clinical charts showed a significant generalized decrease of the T lymphocyte count affecting both CD8^+^ and CD4^+^ T cells with a resulting normal CD4/CD8 ratio in COVID-19 patients (Table 1). In accordance with these results we found a significantly lower T lymphocyte frequency as measured by CD3^+^ frequency in our FACS analysis in samples of COVID-19 patients but not in malaria patients (Figure 1A). A more detailed description of the cohort is given in Table 1.

**Table 1:** Clinical and laboratory characteristics of COVID-19 and malaria patients.

		**COVID-19**	**Malaria**
Patients *n*		20	10
Age in years (range)		54.55 (33–77)	55.5 (36–71)
**Sex**			
Female		5 (25%)	1 (10%)
Male		15 (75%)	9 (90%)
**Comorbidities**			
None		6 (30%)	5 (50%)
Hypertension		6 (30%)	3 (30%)
Diabetes		3 (15%)	2 (20%)
Coronary heart disease		3 (15%)	0 (0%)
Lung disease		5 (25%)	0 (0%)
Cancer		1 (5%)	1 (10%)
Other		10 (50%)	2 (20%)
Days since start of symptoms (range)		9.65 (4–14)	9.6 (2–21)
Hospitalization days (range)		9.9 (1–33)	4.8 (2–15)
Oxygen therapy needed		10 (50%)	0 (0%)
Lung infiltrates		14 (70%)	0 (0%)
Admission to intensive care unit		3 (15%)	1 (10%)
**Disease severity status**			
Mild		6 (25%)	0 (0%)
Moderate		9 (55%)	8 (80%)
Severe		5 (20%)	2 (20%)
**Blood count**			
White blood cell count	3.8–11.0 Mrd/l	5.54 ± 2,24	5.0 ± 0.96
Lymphocyte count	1,000–3,600/μl	1,055.9 ± 456.4	
Hemoglobin	14.0–17.5 g/dl	13.0 ± 1.4	11.83 ± 1.3
Platelets	150–400 Mrd/l	231.75 ± 88.5	108.5 ± 34.1
**T lymphocytes**			
Count	900–2,900/μl	721.2 ± 255.8	n.a.
Percentage	55–84%	67.5 ± 10.7	n.a.
CD4	500–1,350/μl	405.7 ± 151.9	n.a.
CD4%	31–60%	38.4 ± 8.2	n.a.
CD8	290–930/μl	271.1 ± 146.6	n.a.
CD8 %	13–41%	24.7 ± 10.2	n.a.
CD4/CD8 Ratio	0.6–3.6	1.9 ± 0.9	n.a.
Tregs	5.7–10.1%	7.5 ± 3.7	n.a.
**Clinical chemistry**			
CRP	−5 mg/l	74.15 ± 71.2	121.9 ± 65.4
IL-6	<7.0 ng/l	63.02 ± 82.0	n.a.
Ferritin	22.0–322.0 μg/l	740 ± 739	n.a.
Procalcitonin	−0.5 μg/l	0.06 ± 0.06	n.a.
D-dimer	0.21–0.52 mg/l	1.04 ± 0.8	n.a.
Initial Parasitemia %		n.a.	1.995 (1–18)

*Data are expressed as absolute numbers n (n/N) or n (range) or mean with standard deviation*.

### Acute COVID-19 and Malaria Infection Result in a Comparably Elevated Activation Status of the CD8^+^ and CD4^+^ T Cell Populations

For a description of the activation status, we assessed the frequencies of CD69 and HLA-DR/CD38 on CD8^+^ and CD4^+^ T cells in COVID-19 and malaria patients as well as in healthy controls. There were increased levels of CD69 and HLA-DR/CD38 co-expression on CD8^+^ and CD4^+^ T cells in both COVID-19 and malaria patients compared to healthy controls which is characteristic for acute infections (Figure 1B) (18, 37, 38). The gating strategy for CD8^+^ and CD4^+^ T cells is illustrated in Supplementary Figure 3.

**Figure 2:**
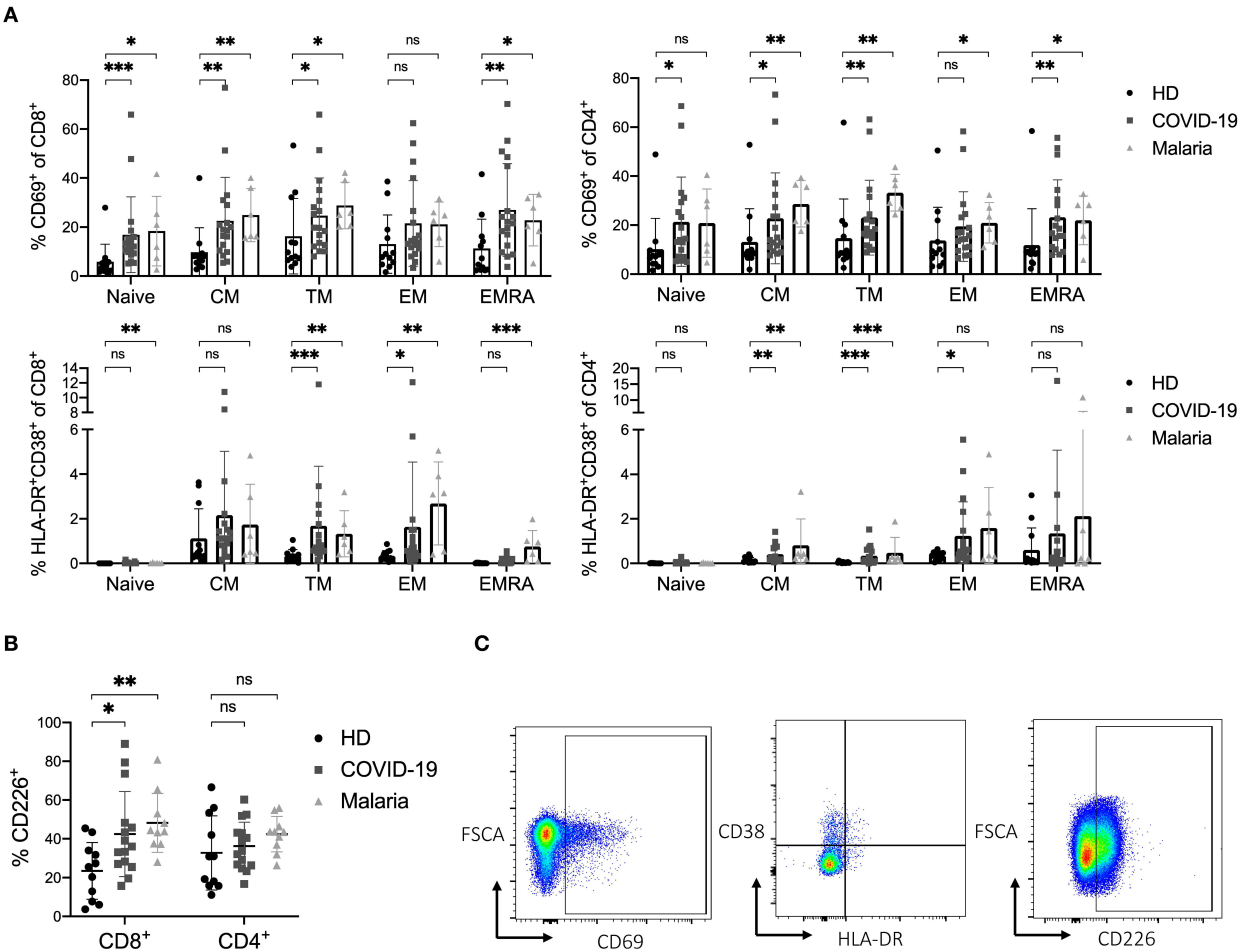
**(A–C)** Activation status of naïve and memory subsets of CD8^+^ and CD4^+^ T cells in COVID-19 and malaria patients compared to healthy donors. **(A)** Frequency of CD69 and co-expression of HLA-DR and CD38 in different CD8^+^ and CD4^+^ T cell differentiation subsets in healthy donors and COVID-19 and malaria patients. **(B)** Frequency of CD226 on CD8^+^ and CD4^+^ T cells in healthy donors and COVID-19 and malaria patients. **(C)** Representative dot plots of PBMC gated on either CD8^+^ or CD4^+^ T cells for T cell activation. *P*-values were calculated by Mann–Whitney test. *P*-values smaller than 0.05 were considered significant, where *, **, and *** indicate *p*-values between 0.01 to 0.05, 0.001 to 0.01, and 0.0001 to 0.001, respectively.

Based on the established differentiation markers CCR7, CD45RO, and CD27 (39), we defined naïve (CCR7^+^CD45RO^−^), central memory CM (CCR7^+^CD45RO^+^), transitional memory TM (CCR7^−^CD45RO^+^CD27^+^), effector memory EM (CCR7^−^CD45RO^+^CD27^−^) and terminal effector memory EMRA (CCR7^−^CD45RO^−^) CD8^+^ and CD4^+^ T cells and assessed the frequency of the activation markers on each subset (39). Overall, we saw an increase of CD8^+^ EM T cells and CD8^+^ and CD4^+^ CM T cells in both COVID-19 and malaria compared to healthy individuals (Figure 1C). Representative dot plots for the gating of naive and memory subsets of CD8^+^ and CD4^+^ T cells can be seen in Figure 1D. CD69 was more frequently expressed on CM, TM, and EMRA CD8^+^ and CD4^+^ T cells in COVID-19 and malaria patients compared to the respective subsets of T cells in healthy individuals. The same statistical trend toward higher CD69 frequencies was evident in the EM subset of CD8^+^ and CD4^+^ T cells in COVID-19 and malaria patients compared to healthy controls (Figure 2A). Looking at the activation as measured by HLA-DR and CD38 expression, we observed an increase of HLA-DR^+^CD38^+^ cells in the TM and EM subset of CD8^+^ T cells and in the CM and TM subset of CD4^+^ T cells in both COVID-19 and malaria patients compared to healthy controls. Of note, CD8^+^ CM, TM, and EM T cells showed particularly high frequencies of HLA-DR^+^CD38^+^ cells in COVID-19 and patients infected with *P. falciparum* (Figure 2A). Together with the overall increase of CM and EM CD8^+^ T cells in COVID-19, this could imply an important role for these cells during the acute infection.

In addition to the analysis of the traditional activation markers, we also looked at the expression of the co-stimulatory receptor CD226 on CD8^+^ and CD4^+^ T cells in COVID-19 and malaria patients compared to healthy individuals. The CD226 molecule enhances cytotoxic effector functions when upregulated on CD8^+^ T cells (14, 40, 41). Indeed, a significantly higher frequency of CD226 was detectable on bulk CD8^+^ T cells in both COVID-19 and malaria compared to healthy individuals. In COVID-19 patients, the TM, EM, and EMRA CD8^+^ T cell subsets showed a higher frequency of CD226 compared to healthy controls, while in malaria patients the upregulation of CD226 was significant across all differentiation subsets compared to healthy individuals which implies a highly activated and functional phenotype of these cells (Figure 2B and Supplementary Figure 4A). Representative dot plots for the gating of CD69, HLA-DR/CD38 and CD226 on CD8^+^ and CD4^+^ T cells are illustrated in Figure 2C.

Overall, we observed a strong activation of T cells in COVID-19 and malaria with an increased expression of both early (CD69^+^) and late (HLA-DR^+^CD38^+^) activation markers as well as the co-stimulatory receptor CD226.

### COVID-19 and Acute Malaria Infection Are Accompanied by Elevated Frequencies of LAG-3 and TIM-3 on CD8^+^ and CD4^+^ T Cells

**Figure 3:**
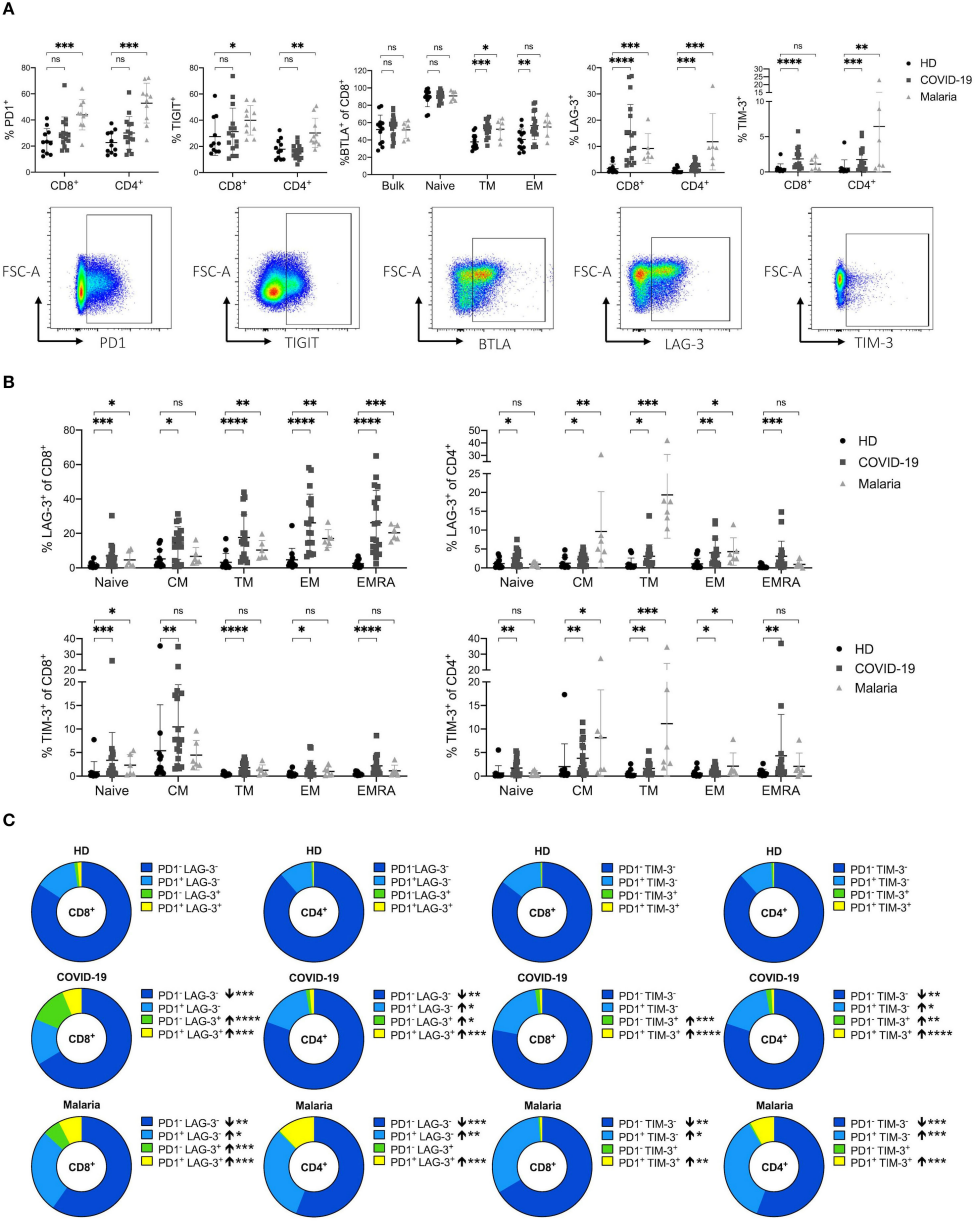
**(A–C)** Frequency and co-expression of inhibitory receptors on CD8^+^ and CD4^+^ T cells in COVID-19 and malaria patients compared to healthy donors. **(A)** Frequency of co-inhibitory receptors PD1, TIGIT, LAG-3, TIM-3, and BTLA on CD8^+^ and CD4^+^ T cells in healthy donors and COVID-19 and malaria patients. Representative dot plots of the PD1, TIGIT, TIM-3, LAG-3, and BTLA frequency gated on either CD8^+^ or CD4^+^ T cells. **(B)** Frequency of LAG-3 and TIM-3 on the CD8^+^ and CD4^+^ T cell differentiation subsets in healthy donors and COVID-19 and malaria patients. **(C)** Co-expression of PD1/LAG-3 and PD1/TIM-3 illustrated in donut charts showing the mean frequency of CD8^+^ and CD4^+^ T cells in healthy donors and COVID-19 and malaria patients. Frequencies in COVID-19 and malaria were compared with HD. *P*-values were calculated by Mann–Whitney test. *P*-values smaller than 0.05 were considered significant, where *, **, ***, and **** indicate *p*-values between 0.01 to 0.05, 0.001 to 0.01, 0.0001 to 0.001 and <0.0001 respectively.

Next, we examined the expression of the inhibitory receptors PD1, TIGIT, LAG-3, TIM-3, and BTLA of T cells in COVID-19 and malaria compared to healthy controls. Previous research has established inhibitory receptors to play a critical role in T cell exhaustion in chronic infections after prolonged antigen exposure (14, 15, 42, 43). However, inhibitory receptors also have important functions and are transiently expressed during acute infections (17, 24, 44–46). Many studies have described an increased expression of inhibitory receptors on both CD8^+^ and CD4^+^ T cells during malaria infection (26, 27, 47, 48). Whether this increase has a beneficial or damaging effect, however, is not yet fully understood. To see whether similar effects can be observed in COVID-19, we assessed the frequencies of the co-inhibitory receptors PD1, TIGIT, BTLA, LAG-3, and TIM-3 on CD8^+^ and CD4^+^ T cells in COVID-19 and malaria patients and compared them with healthy individuals (Figure 3A). We also assessed the expression of these inhibitory receptors on the different memory subsets to detect potential influences on the expression due to a change in subset distribution (Figure 3B and Supplementary Figure 4).

A trend toward higher PD1 expression on CD8^+^ and CD4^+^ T cells in COVID-19 could be observed although with no statistical significance (Figure 3A). However, malaria patients showed a strong upregulation of PD1 on both CD8^+^ and CD4^+^ T cells (Figure 3A). This increase of PD1 on T cells in malaria was significant across all memory subsets of CD4^+^ T cells and on naïve and EMRA CD8^+^ T cells (Supplementary Figure 4B). The mean fluorescence intensity (MFI) of PD1 showed an increased expression of PD1 on bulk CD8^+^ T cells in COVID-19 and malaria patients compared to healthy individuals. In contrast, while we could not see any difference in the PD1 MFI on CD4^+^ T cells between COVID-19 patients and healthy donors, malaria patients showed a significantly increased PD1 MFI on bulk, naïve, and all CD4^+^ memory T cell subsets compared to healthy donors. Interestingly, the EM subset of CD8^+^ T cells, which presumably includes a high proportion of SARS-CoV-2-specific T cells (10), showed a comparable PD1 MFI in healthy donors and COVID-19 and malaria patients (Supplementary Figure 4C).

We did not detect increased TIGIT expression on bulk T cells in COVID-19 patients compared to healthy controls (Figure 3A). The EM CD4^+^ T cell subset in COVID-19 even showed a significant decrease of TIGIT expression. The same trend was evident on CD8^+^ EM T cells although this trend did not reach statistical significance. TIGIT expression in malaria patients was significantly increased on bulk CD8^+^ and CD4^+^ T cell populations. However, when looking at the expression pattern of TIGIT on different memory subsets, only TM CD4^+^ T cells showed a significantly higher TIGIT frequency (Supplementary Figure 4D). Overall it seems that in acute COVID-19 infection there is a trend toward a decreased TIGIT frequency on T cells compared to healthy donors while T cells in acute malaria patients generally showed a trend toward higher TIGIT frequencies compared to healthy individuals.

BTLA inhibits lymphocyte effector functions during immune response, similar to PD1 (49). It is well-established that BTLA expression is downregulated during CD8^+^ and CD4^+^ T cell differentiation, with the highest expression on naïve T cells (49). Indeed, a lower BTLA frequency in memory compared to naive subsets of CD8^+^ and CD4^+^ T cells could be detected in COVID-19 and malaria patients as well as healthy donors (Figure 3A). However, the decrease of BTLA on CD8^+^ T cell subsets detected in both, COVID-19 and malaria, was not as strong as in healthy subjects. We observed a significantly higher expression of BTLA on TM and EM CD8^+^ T cells in COVID-19 and malaria patients compared to healthy donors (Figure 3A).

Lastly, we looked at the expression of LAG-3 and TIM-3. Both inhibitory receptors have previously been described to be upregulated during acute malaria infection with complex effects on T cell function (26). Accordingly, we observed a significant increase of LAG-3 and TIM-3 on CD8^+^ and CD4^+^ T cells in malaria patients when compared to healthy donors. In COVID-19 patients we saw a similar upregulation of LAG-3 and TIM-3 on T cells of even higher frequencies on CD8^+^ T cells (Figure 3A). A significant increase of LAG-3 and TIM-3 on both CD8^+^ and CD4^+^ T cells across all subpopulations in COVID-19 patients could be detected (Figure 3B).

To further characterize the expression pattern of LAG-3 and TIM-3 we examined the co-expression of PD1/LAG-3 and PD1/TIM-3 on CD8^+^ and CD4^+^ T cells of COVID-19 and malaria patients compared to healthy controls (Figure 3C). Interestingly, we found differences in the distribution of these receptors, not only when comparing T cells of COVID-19 and malaria patients with T cells of healthy donors, but also when comparing COVID-19 with malaria. CD8^+^ and CD4^+^ T cells of COVID-19 patients showed high frequencies of single-positive PD1^−^LAG-3^+^ and PD1^−^TIM-3^+^ CD8^+^ and CD4^+^ T cells with a significant increase compared to healthy controls. However, in PBMC of malaria patients we only observed an increase of PD1^−^LAG-3^+^ CD8^+^ T cells when compared to the frequency of PD1^−^LAG-3^+^ CD8^+^ and CD4^+^ T cells in healthy controls. Double-positive PD1^+^LAG-3^+^ and PD1^+^TIM-3^+^ cells were increased in both COVID-19 and malaria when compared with healthy donors. Looking at the single-positive PD1^+^LAG-3^−^ and PD1^+^TIM-3^−^ cells we found no difference between COVID-19 patients and healthy donors in the CD8^+^ T cell compartment. Only CD4^+^ T cells in COVID-19 showed a higher frequency of PD1^+^LAG-3^−^ and PD1^+^TIM-3^−^ T cells compared to healthy donors although the frequency of these cells was highest in malaria with a significant increase of both PD1^+^LAG-3^−^ and PD1^+^TIM-3^−^ on CD8^+^ and CD4^+^ T cells compared to healthy controls. Overall, we saw a shift toward double-positive PD1^+^LAG-3^+^ and PD1^+^TIM-3^+^ and single-positive PD1^−^LAG-3^+^ and PD1^−^TIM-3^+^ cells in the CD8^+^ T cell subset of COVID-19 patients whereas in malaria, the distribution changed more toward double-positive PD1^+^LAG-3^+^ and PD1^+^TIM-3^+^ and single-positive PD1^+^LAG-3^−^ and PD1^+^TIM-3^−^ T cells. Lastly, we looked for PD1^+^LAG-3^+^TIM-3^+^ T cells. However, the frequency of these PD1^+^LAG-3^+^TIM-3^+^ T cells was comparably low ranging from only 0.013–0.76% of CD8^+^ T cells in COVID-19 (data not shown).

Overall, we saw a strong increase of LAG-3 and TIM-3 expression on T cells in COVID-19 and malaria. PD1 was only slightly increased on T cells in COVID-19 patients while the frequency of TIGIT on T cells did not differ between COVID-19 patients and healthy controls. In malaria we observed an upregulation of both PD1 and TIGIT on CD8^+^ and CD4^+^ T cells compared to healthy donors.

### Co-expression of PD1, LAG-3, and TIM-3 on T Cells Depends on the Degree of T Cell Activation

**Figure 4:**
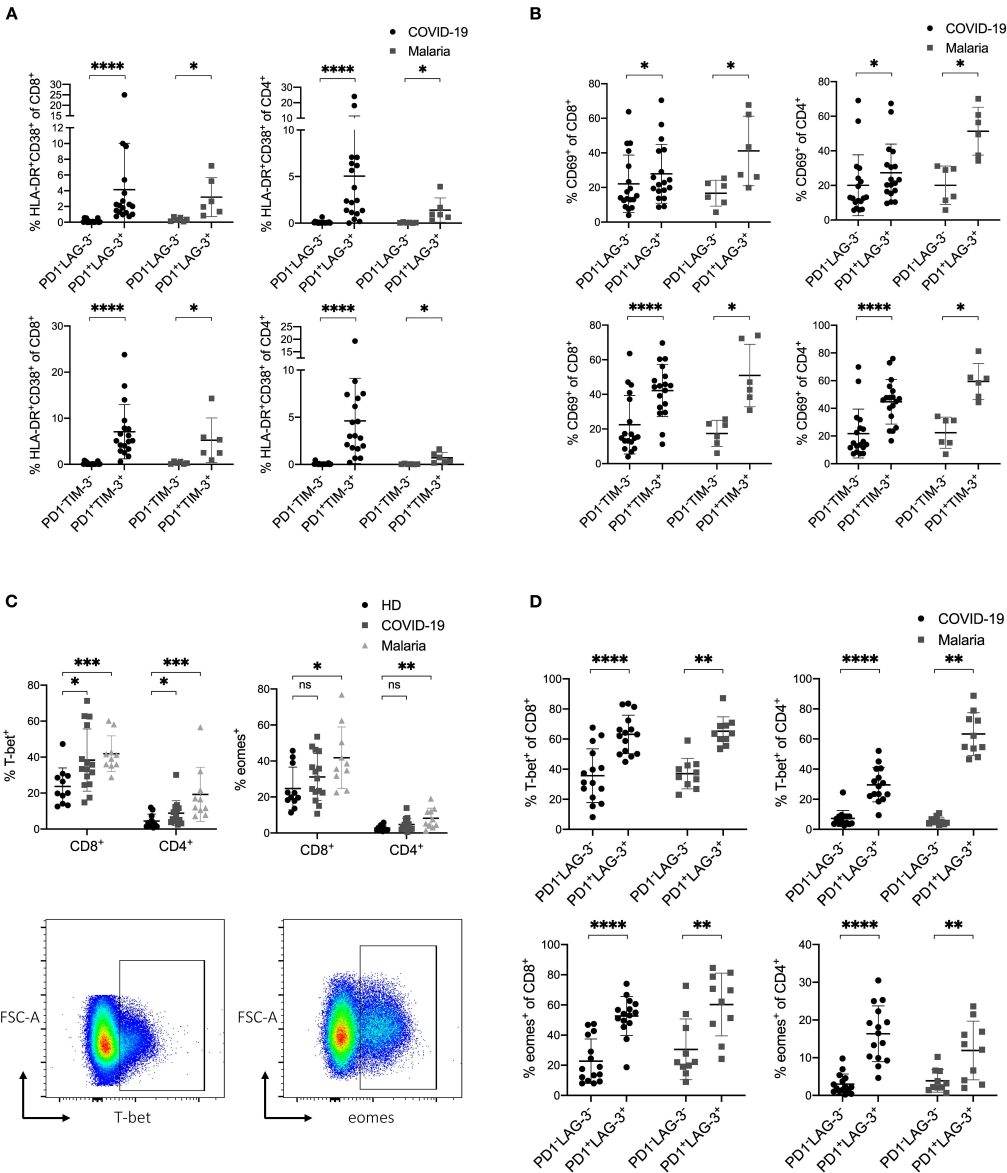
**(A–D)** Expression profile of inhibitory and stimulatory receptors as well as the transcription factors T-bet and eomes in COVID-19 and malaria patients. **(A)** Frequency of HLA-DR/CD38 on PD1^−^LAG-3^−^, PD1^−^TIM-3^−^, PD1^+^LAG-3^+^, and PD1^+^TIM-3^+^ CD8^+^ and CD4^+^ T cells in COVID-19 and malaria patients. **(B)** Frequency of CD69 on PD1^−^LAG-3^−^, PD1^−^TIM-3^−^, PD1^+^LAG-3^+^ and PD1^+^TIM-3^+^ CD8^+^ and CD4^+^ T cells in COVID-19 and malaria patients. **(C)** Frequency of T-bet and eomes in CD8^+^ and CD4^+^ T cells in healthy donors and COVID-19 and malaria patients. Representative dot plots of the T-bet and eomes frequency gated on either CD8^+^ or CD4^+^ T cells. **(D)** Frequency of T-bet and eomes in PD1^−^LAG-3^−^ and PD1^+^LAG-3^+^ CD8^+^ and CD4^+^ T cells in COVID-19 and malaria patients. *P*-values were calculated by Wilcoxon test for paired analysis and Mann–Whitney test for unpaired analysis. *P*-values smaller than 0.05 were considered significant, where *, **, ***, and **** indicate *p*-values between 0.01 to 0.05, 0.001 to 0.01, 0.0001 to 0.001 and <0.0001 respectively.

T cells of patients with COVID-19 and acute malaria showed high frequencies of the activation markers CD69 and HLA-DR/CD38 as well as high frequencies of the inhibitory receptors LAG-3 and TIM-3. To characterize whether stimulatory and inhibitory receptors are co-expressed on, or limited to distinct T cells we analyzed the expression of CD69 and the co-expression of HLA-DR/CD38 on PD1^−^LAG-3^−^ and PD1^−^TIM-3^−^ T cells and compared it to the expression on double-positive PD1^+^LAG-3^+^ and PD1^+^TIM-3^+^ T cells in COVID-19 and malaria patients. Double-negative T cells did not co-express HLA-DR and CD38 while double-positive T cells showed high frequencies of HLA-DR^+^CD38^+^ CD8^+^ and CD4^+^ T cells (Figure 4A). This upregulation of HLA-DR/CD38 on T cells co-expressing PD1 and LAG-3 and PD1 and TIM-3 could be observed on both CD8^+^ and CD4^+^ T cells in COVID-19 and malaria patients. Similarly, the frequency of CD69 was lower on PD1^−^LAG-3^−^ and PD1^−^TIM-3^−^ CD8^+^ and CD4^+^ T cells compared to the expression on double-positive T cells in COVID-19 and malaria patients (Figure 4B). In summary, the expression of activation markers (CD69, HLA-DR, CD38) on T cells was closely linked to the co-expression of inhibitory receptors (PD1, LAG-3, TIM-3) on T cells in COVID-19 and malaria patients.

### Upregulation of T-bet and Eomes in T Cells That Co-express Inhibitory Receptors in COVID-19 and Acute Malaria Infection

In a next step, we analyzed the transcription factors T-bet and eomes in COVID-19 and malaria patients. For this, we stained PBMC with the transcription factors T-bet and eomes and analyzed their expression in T cells in relation to the co-inhibitory receptors PD1 and LAG-3. The transcription factors T-bet and eomes have important functions in the immune response to acute infections and are upregulated to preserve cytotoxic effector functions in T cells (50–54). In chronic settings it has been shown that exhaustion and functional impairment of T cells is accompanied by an upregulation of eomes and downregulation of T-bet in exhausted T cells (22, 55, 56). Looking at the expression of T-bet and eomes in CD8^+^ and CD4^+^ T cells in COVID-19 and malaria patients we saw an upregulation of T-bet in COVID-19 and an upregulation of both T-bet and eomes in malaria (Figure 4C). Next, we aimed to look at T-bet and eomes in the context of the co-inhibitory receptors PD1 and LAG-3. For this, we compared the expression of T-bet and eomes in PD1^+^LAG-3^+^ T cells to that in PD1^−^LAG-3^−^ T cells in COVID-19 and malaria (Figure 4D). Similar to the expression of activation markers on these cells, T-bet and eomes were both more frequently expressed in PD1^+^LAG-3^+^ T cells than in their double-negative counterparts. Altogether, the simultaneous upregulation of T-bet and eomes in T cells co-expressing PD1 and LAG-3 further supports the notion that an upregulation of co-inhibitory receptors can be seen as a result of the overall immune activation during COVID-19 and acute malaria infection.

Additionally, we examined the frequency of CD127 expression on CD8^+^ T cells in COVID-19 and malaria patients in relation to the expression of the co-inhibitory receptors PD1 and LAG-3. CD127 as part of the IL-7 receptor is hugely important for the survival and differentiation of T cells and is involved in memory development (57, 58). CD127^low^ T cells are considered short-lived effector cells that undergo apoptosis after antigen clearance (59). In some viral infections such as HIV and HCV the downregulation of CD127 on bulk CD8^+^ T cells has been demonstrated to have damaging effects on viral control and promote T cell exhaustion (60, 61). Bulk CD8^+^ T cells of COVID-19 and malaria patients, however, showed no difference in CD127 expression compared with healthy individuals (Supplementary Figure 5A). CD127 on PD1^+^LAG-3^+^ CD8^+^ Tells in COVID-19 and malaria was downregulated compared to their double-negative PD1^−^LAG-3^−^ counterparts (Supplementary Figure 5B).

### Detection of High Expression of LAG-3 and TIM-3 on T Cells in Patients With Severe COVID-19 and Rapid Normalization After Clinical Recovery

**Figure 5:**
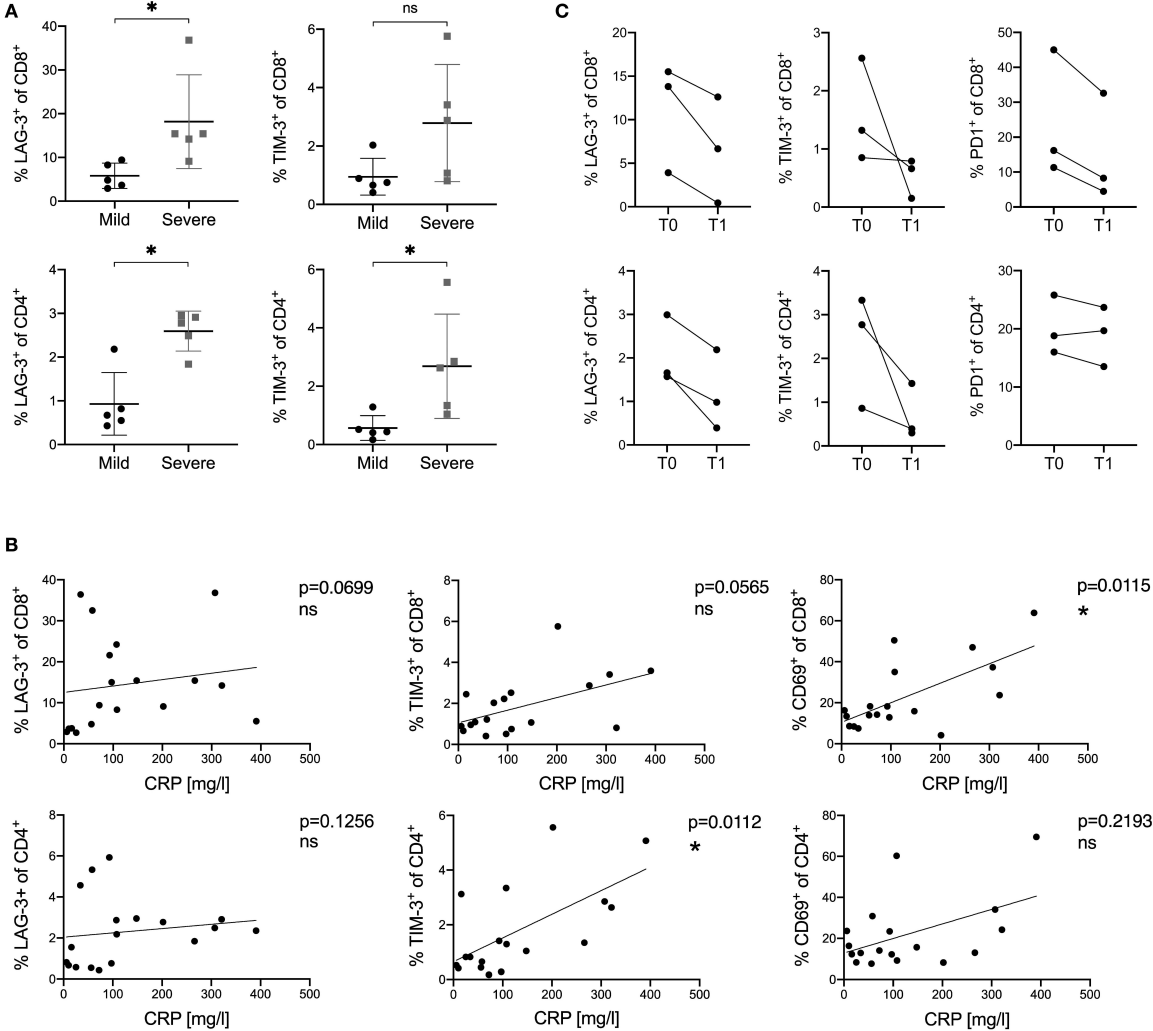
**(A–C)** Correlation of the expression of inhibitory and stimulatory receptors on T cells with disease course and CRP levels in COVID-19 patients. **(A)** Comparison of LAG-3 and TIM-3 frequency on CD8^+^ and CD4^+^ T cells between mild and severe COVID-19 patients. **(B)** Correlations between CRP levels and frequencies of LAG-3, TIM-3, and CD69 on CD8^+^ and CD4^+^ T cells in COVID-19 patients. **(C)** Frequency of PD1, LAG-3, and TIM-3 on CD8^+^ and CD4^+^ T cells measured at different time points in three COVID-19 patients. T0 indicates the first sample date. T1 indicates the second sample date (13, 15, or 20 days after T0). *P*-values were calculated by Mann–Whitney test. For bivariate correlation analysis the Spearman correlation was applied. *P*-values smaller than 0.05 were considered significant, where * indicate p-values between 0.01 to 0.05.

To further evaluate whether the expression of LAG-3 and TIM-3 on T cells was related to the severity and disease course in COVID-19, we sub-stratified patients into a mild and a severe group (Table 1). Patients with mild disease course were defined to have a maximum hospital stay of five days and absence of any complications. Severe disease course was defined as hospital stay longer than 20 days, oxygen saturation lower than 85%, transfer to the ICU or death. According to this definition, six patients fitted into the mild group and five patients fitted into the severe group. Next, we compared the activation and inhibitory marker expression on T cells between these two groups. Interestingly, we saw a higher expression of LAG-3 and TIM-3 on CD8^+^ and CD4^+^ T cells in patients with a severe disease course compared to patients with a mild disease course (Figure 5A). However, there was no significantly higher expression of other co-inhibitory receptors on T cells in the severe group compared to the mild group (Supplementary Figure 6A). Additionally, we also compared the peak levels of the clinical inflammation parameters C-reactive protein (CRP), IL-6, or ferritin with the expression of activation and inhibitory receptors on CD8^+^ and CD4^+^ T cells in COVID-19. CD69 on CD8^+^ T cells and TIM-3 on CD4^+^ T cells correlated with CRP levels (Figure 5B). A similar trend of correlation between CRP levels and the frequency of TIM-3 on CD8^+^ T cells could be observed. IL-6 and ferritin levels did not correlate with the expression of co-inhibitory molecules (data not shown).

Finally, we investigated the expression kinetics of inhibitory receptors during the course and recovery of COVID-19 infection. For this, PBMC of three COVID-19 patients were collected at a second time point on day 13, 15, or 20 after the first sample date. All three patients were discharged in good health from the hospital shortly after the second sample collection. Frequencies of inhibitory receptor expression on CD8^+^ and CD4^+^ T cells were analyzed and compared to those measured at the first time point. A rapid decrease and normalization in all three patients for LAG3, TIM3, and PD1 expression on CD8^+^ and CD4^+^ T cells was observed, independently of the individual expression level measured at the first time point (Figure 5C).

## Discussion

One of the main results of this cross-sectional side-by-side comparison of co-inhibitory and co-stimulatory receptors on T cells isolated from PBMC of patients with acute COVID-19 and malaria infection was the comparable T cell activation and phenotype observed, following SARS-CoV-2 and *P. falciparum* infection. Of interest, a strong upregulation of LAG-3 and TIM-3 on T cells in both COVID-19 and malaria patients was linked to the co-expression of activation markers and correlated with disease course (Figures 3–5) (26). LAG-3 and TIM-3 are co-inhibitory receptors with multiple functions in modulating pro-inflammatory T cell responses (62).

Elevation and sustained co-expression of inhibitory receptors such as PD1, LAG-3, TIM-3, CTLA4, and BTLA is the hallmark of exhausted T cells (14, 20, 42). This unique T cell population is the result of persisting antigen stimulation found in cancer and chronic infections. Previous studies of T cells in malaria have shown that the upregulation of co-inhibitory molecules such as LAG-3 and TIM-3 in response to acute infection can have both beneficial and detrimental effects (29–32). It is still highly debated whether T cell exhaustion can occur during early malaria infection or other acute viral infections since the expression of inhibitory receptors is also induced through T cell activation and differentiation (17, 24, 27, 63). Legat et al. showed that stimulating human CD8^+^ T cells induced a strong and rapid upregulation of inhibitory receptors after 24 h while cytokine production was not necessarily affected (17). Additionally, in a murine LCMV model it was demonstrated that irreversible T cell exhaustion occurred only after about 30 days of infection (25).

Our data demonstrate a strong upregulation of LAG-3 and TIM-3 on T cells in COVID-19 and malaria patients across all differentiation stages. In COVID-19 patients we found high frequencies of single-positive PD1^−^LAG-3^+^ and PD1^−^TIM-3^+^ CD8^+^ and CD4^+^ T cells and fewer double-positive PD1^+^LAG-3^+^ and PD1^+^TIM-3^+^ T cells. However, in malaria patients we found high levels of single-positive PD1^+^LAG-3^−^ and PD1^+^TIM-3^−^ as well as high levels of double-positive PD1^+^LAG-3^+^ and PD1^+^TIM-3^+^ CD8^+^ and CD4^+^ T cells. Triple-positive PD1^+^LAG-3^+^TIM-3^+^ T cells were nearly absent in both COVID-19 and malaria patients. Double-positive cells were highly activated compared to their double-negative counterparts and showed increased levels of the transcription factors T-bet and eomes in COVID-19 and malaria. A previous study by Niu et al. demonstrated that the expression of PD1 and LAG-3 on T cells in patients with acute sepsis had a regulatory impact on cytokine production (64). The degree of inhibition was dependent on the expression pattern of PD1 and LAG-3 on T cells. In comparison, single-positive PD1^−^LAG-3^+^ T cells showed the highest cytokine production followed by single-positive PD1^+^LAG-3^−^ T cells while double-positive PD1^+^LAG-3^+^ T cells produced the least amount of cytokines (64).

Overall, when comparing the activation and expression signatures of different co-inhibitory receptors in COVID-19 and malaria patients we found relatively subtle differences between T cells of patients with either infection. The activation markers CD69 and HLA-DR/CD38 were elevated on T cells in COVID-19 and malaria patients. The upregulation of the co-inhibitory receptors LAG-3 and TIM-3 was more pronounced on CD8^+^ T cells in COVID-19 patients while malaria patients showed especially high expression of LAG-3 and TIM-3 on CD4^+^ T cells. In line with this, acute malaria infection has been shown to result in a strong polarization of CD4^+^ T cells (26) while it can be speculated that there seems to be a polarization toward CD8^+^ T cells in COVID-19. Furthermore, T cells of malaria patients showed even higher frequencies of PD1 and TIGIT than COVID-19 patients and healthy donors (Figure 3A). Overall, our data show a rather similar T cell signature of co-inhibitory molecules in COVID-19 and malaria, an infection in which the upregulation of co-inhibitory receptors is a result of their strong activation (26, 27). However, acute malaria generally seemed to result in an upregulation and co-expression of a broader range of co-inhibitory receptors on T cells (Figure 3A).

Importantly, we also observed a strong upregulation of the transcription factors T-bet and eomes in T cells co-expressing inhibitory receptors. T-bet and eomes are important mediators during acute infections and promote effector functions and cytotoxicity in CD8^+^ and CD4^+^ T cells (50–53). T-bet drives the expansion of cytokine producing Th1 cells and inhibits the formation of Th2 and Th17 cells (50, 51). In this context T-bet has also been shown to repress the expression of PD1 and counter-regulate the Th1 response by inducing TIM-3 on CD4^+^ T cells. In CD8^+^ T cells T-bet promotes cytotoxicity by inducing the production of granzyme B and perforin (50, 52). Eomes is also found in both CD8^+^ and CD4^+^ T cells during acute infection. In CD4^+^ T cells eomes can either induce the production of IFN-γ by Th1 cells or promote Tr1 cells by driving IL-10 production although the exact mechanisms underlying this are not yet fully understood (65, 66). In CD8^+^ T cells eomes is needed for a T-bet independent induction of IFN-γ and later during infection drives the formation of memory cells by aiding the responsiveness to IL-15 (52). Regarding T cell exhaustion, T-bet and eomes have distinct roles compared to those in effector and memory T cells. Exhausted T cells express high levels of eomes and decreased expression of T-bet (22, 56, 67). We observed an increase of T-bet in bulk CD8^+^ and CD4^+^ T cells and high frequencies of both T-bet and eomes in CD8^+^ and CD4^+^ T cells co-expressing PD1 and LAG-3 indicating strong effector functions in these cells. Further and more comprehensive studies of the transcription factor profile of T cells in COVID-19 and malaria are needed to understand and interpret the expression pattern of inhibitory receptors.

Recently, the CD226/TIGIT axis has been identified as an important regulator in anti-tumor and anti-viral T cell responses (14). TIGIT expression on CD8^+^ and CD4^+^ T cells in COVID-19 patients did not differ greatly from healthy individuals. We even observed a downregulation of TIGIT on some T cell subsets (Supplementary Figure 4B). Considering that TIGIT and CD226 compete for the same ligand CD155 on APCs and binding of CD226 to this ligand promotes cytotoxicity in CD8^+^ T cells the simultaneous increase of CD226 and decrease of TIGIT expression could benefit CD8^+^ T cells to maintain cytotoxic effector functions in COVID-19 (40, 68). In malaria patients we observed an increase of TIGIT on bulk CD8^+^ and CD4^+^ T cells. However, looking at the different T cell subsets no significant changes could be seen which indicates that the increase in TIGIT expression might be linked to the differentiation status and change in distribution away from naïve toward effector cells. However, the overall frequency of TIGIT^+^ T cells in malaria and COVID-19 patients was lower than the frequency observed in chronic settings such as HCV (14).

Expression of LAG-3 and TIM-3 on T cells in COVID-19 patients was closely related to disease course with higher levels detectable in severe patients compared to the mild patient group. Towards recovery of COVID-19 infection, the expression of inhibitory receptors on T cells decreased rapidly. Taken together, our results do not hint towards the development of terminal T cell exhaustion during acute COVID-19 infection. However, whether the upregulation of inhibitory receptors on T cells in COVID-19 has detrimental or beneficial effects needs to be further investigated. Moreover, we only analyzed the bulk expression levels of co-inhibitory molecules of T cells and pathogen-specific T cells might show a different pattern.

On the same note, blockade of the inhibitory receptors LAG-3 and TIM-3 as a therapeutic strategy in COVID-19 or malaria must be critically evaluated (69). On the one hand, it could enhance the effector T cell response to SARS-CoV-2 infection, on the other hand, such an intervention could also increase hyperinflammation. Interestingly, Robilotti et al. describe that therapy with immune checkpoint inhibitors correlated with a more severe outcome in COVID-19 patients (69). Interestingly, two patients of our COVID-19 cohort were previously treated with biologicals (Rituximab and Pembrolizumab) but neither patient showed a severe disease course, increased hyperinflammation or differing T cell pattern in terms of activation, differentiation, or expression of co-inhibitory molecules (data not shown). Nonetheless, study of the immune response following SARS-CoV-2 infection in patients receiving immunomodulatory therapy can give us further insight into the role and function of different immune cells.

Disease progression and adverse outcome in COVID-19 seemed to be associated with the degree of hyperactivation leading to cytokine storm and lymphopenia (4). The question remains, whether and in how far T effector cells are involved in the pathophysiology and hyperinflammation of COVID-19. The so-called cytokine storm is known from other infections such as severe acute respiratory syndrome (SARS) and ebola (70, 71). High levels of cytokines like IL-6, or Interferon-y could be measured in the serum of COVID-19 patients, hinting toward a dysbalanced, overreacting immune system (4). The underlying mechanisms and driving forces of this cytokine storm in COVID-19 remain to be fully understood. However, from our results and findings in initial COVID-19 studies, it appears that T cells do not play a direct role in the development of hyperinflammation (10, 72). The main source of IL-6 does not appear to be T cells but rather monocytes and macrophages (10, 73). It can even be speculated whether the upregulation of LAG-3 and TIM-3 is a reaction of the immune system to counteract systemic hyperinflammation. Interestingly, a study on ebola has demonstrated that fatal ebola infections were associated with higher expression of CTLA4 and PD1 on CD8^+^ and CD4^+^ T cells and was accompanied by strong inflammation while ebola patients who survived showed less inflammation markers and lower CTLA4 and PD1 expression on T cells (74). In accordance, our data show a correlation between disease severity of COVID-19 and expression of inhibitory receptors like LAG-3 and TIM-3 on T cells.

Our study was limited by the relatively small and heterogeneous cohort and we assessed only few patients longitudinally. In order to further investigate the exhaustive state of the SARS-CoV-2-specific T cell response in COVID-19, intracellular cytokine assays (ICS) after antigen-specific short term stimulation, MHC class I and class II multimer-staining and further functional assays in larger prospective cohorts with broader variations of the clinical course and worse outcome are needed to understand the role and function of antigen-specific cells. Additionally, functional T cell subsets such as follicular helper cells, Th17, and regulatory T cells need to be characterized in parallel. Of note, the frequency of peripheral Tregs as measured by clinical immunology did not differ between patients with COVID-19 and healthy individuals (Table 1). In our study, we examined the phenotype of peripheral CD8^+^ and CD4^+^ T cells. It has been shown that the expression pattern of co-inhibitory receptors on T cells depends on their localization (75). T cells in the peripheral blood can greatly differ in their expression pattern to those in the tissue (76). Considering this, further studies that examine T cells in the lung, liver, and other organs in COVID-19 and malaria are needed.

The direct comparison of the T cell response between an acute viral and an acute parasitic infection does not seem self-evident. However, there are only few acute infections which are accompanied by hyperinflammation with high levels of pro-inflammatory cytokines and in which the T cell response is regulated by a strong upregulation of a broad spectrum of co-inhibitory receptors, such as ebola, SARS, MERS, and malaria (28, 71). Interestingly, we found relatively minor differences of the T cell signature and co-expression of co-inhibitory molecules of T cells in the direct comparison of patients with COVID-19 and acute malaria supporting the notion that co-inhibitory molecules have a dual function, with potentially beneficial but also detrimental effects on T cell function (26, 29, 31, 32). Our data suggest that there is a common pattern of T cell regulation in acute infections and that the up-regulation and co-expression of co-inhibitory molecules is as a consequence of their strong activation. Nonetheless, comparisons of the T cell phenotype and functional immune response with other acute viral respiratory infections like influenza are needed in future studies.

In summary, comparing the expression of different co-inhibitory molecules of CD8^+^ and CD4^+^ T cells in COVID-19 vs. malaria there is a transient increase of the expression of certain inhibitory receptors like LAG-3 and TIM-3 in COVID-19 in the overall context of acute immune activation. During recovery, a rapid normalization of these inhibitory receptors could be observed. The results of this study will be the basis for further analysis regarding T cells and their potential role for immune pathogenesis during COVID-19 infection.

## Data Availability Statement

The datasets generated for this study are available on request to the corresponding author.

## Ethics Statement

The studies involving human participants were reviewed and approved by Ärztekammer Hamburg (PV4238, PV4780, and PV7298). The patients/participants provided their written informed consent to participate in this study.

## Author Contributions

MH, SS, TJ, and JS designed the study. MH, SS, NW, MW, TJ, and JS designed the panels. MH, SS, NW, RW, and MW conducted experiments. MH and SS analyzed the data. JS, TB, and MR recruited the patients. JS supervised the study at all stages. MH, SS, TJ, and JS wrote the first draft. JS and AL gave institutional support. All authors reviewed the manuscript and gave important input.

## Conflict of Interest

The authors declare that the research was conducted in the absence of any commercial or financial relationships that could be construed as a potential conflict of interest.

## References

[1] Grifoni A Sidney J Zhang Y Scheuermann RH Peters B Sette A. A sequence homology and bioinformatic approach can predict candidate targets for immune responses to SARS-CoV-2. Cell Host Microbe. (2020) 27:671–80.e2. 10.1016/j.chom.2020.03.002 32183941 PMC7142693

[2] Wu F Zhao S Yu B Chen YM Wang W Song ZG. A new coronavirus associated with human respiratory disease in China. Nature. (2020) 579:265–9. 10.1038/s41586-020-2008-3 32015508 PMC7094943

[3] Okba NMA Müller MA Li W Wang C GeurtsvanKessel CH Corman VM. Severe acute respiratory syndrome coronavirus 2-specific antibody responses in coronavirus disease patients. Emerg Infect Dis. (2020) 26:1478–88. 10.3201/eid2607.200841 32267220 PMC7323511

[4] Huang C Wang Y Li X Ren L Zhao J Hu Y. Clinical features of patients infected with 2019 novel coronavirus in Wuhan, China. Lancet. (2020) 395:497–506. 10.1016/S0140-6736(20)30183-5 31986264 PMC7159299

[5] Chen G Wu D Guo W Cao Y Huang D Wang H. Clinical and immunological features of severe and moderate coronavirus disease 2019. J Clin Invest. (2020) 130:2620–9. 10.1172/JCI137244 32217835 PMC7190990

[6] Li LQ Huang T Wang YQ Wang ZP Liang Y Huang TB. COVID-19 patients' clinical characteristics, discharge rate, and fatality rate of meta-analysis. J Med Virol. (2020) 92:577–83. 10.1002/jmv.25757 32162702 PMC7228329

[7] Zhang B Zhou X Qiu Y Song Y Feng F Feng J. Clinical characteristics of 82 cases of death from COVID-19. PLoS One. (2020) 15:e0235458. 10.1371/journal.pone.0235458 32645044 PMC7347130

[8] Shi Y Wang Y Shao C Huang J Gan J Huang X. COVID-19 infection: the perspectives on immune responses. Cell Death Differ. (2020) 27:1451–4. 10.1038/s41418-020-0530-3 32205856 PMC7091918

[9] Diao B Wang C Tan Y Chen X Liu Y Ning L. Reduction and functional exhaustion of T cells in patients with coronavirus disease 2019 (COVID-19). Front Immunol. (2020) 11:827. 10.3389/fimmu.2020.00827 32425950 PMC7205903

[10] Weiskopf D Schmitz KS Raadsen MP Grifoni A Okba NMA Endeman H. Phenotype and kinetics of SARS-CoV-2-specific T cells in COVID-19 patients with acute respiratory distress syndrome. Sci Immunol. (2020) 5:eabd2071. 10.1101/2020.04.11.20062349 32591408 PMC7319493

[11] Wang J Jiang M Chen X Montaner LJ. Cytokine storm and leukocyte changes in mild versus severe SARS-CoV-2 infection: review of 3939 COVID-19 patients in China and emerging pathogenesis and therapy concepts. J Leukoc Biol. (2020) 108:17–41. 10.1002/JLB.3COVR0520-272R 32534467 PMC7323250

[12] Channappanavar R Fett C Zhao J Meyerholz DK Perlman S. Virus-specific memory CD8 T cells provide substantial protection from lethal severe acute respiratory syndrome coronavirus infection. J Virol. (2014) 88:11034–44. 10.1128/JVI.01505-14 25056892 PMC4178831

[13] Taylor PM Askonas BA. Influenza nucleoprotein-specific cytotoxic T-cell clones are protective *in vivo*. Immunology. (1986) 58:417–20. 2426185 PMC1453480

[14] Ackermann C Smits M Woost R Eberhard JM Peine S Kummer S. HCV-specific CD4+ T cells of patients with acute and chronic HCV infection display high expression of TIGIT and other co-inhibitory molecules. Sci Rep. (2019) 9:10624. 10.1038/s41598-019-47024-8 31337800 PMC6650447

[15] Day CL Kaufmann DE Kiepiela P Brown JA Moodley ES Reddy S. PD-1 expression on HIV-specific T cells is associated with T-cell exhaustion and disease progression. Nature. (2006) 443:350–4. 10.1038/nature05115 16921384

[16] Chen DY Wolski D Aneja J Matsubara L Robilotti B Hauck G. Hepatitis C virus-specific CD4+ T cell phenotype and function in different infection outcomes. J Clin Invest. (2020) 130:768–73. 10.1172/JCI126277 31904582 PMC6994113

[17] Legat A Speiser DE Pircher H Zehn D Fuertes Marraco SA. Inhibitory receptor expression depends more dominantly on differentiation and activation than “exhaustion” of human CD8 T cells. Front Immunol. (2013) 4:455. 10.3389/fimmu.2013.00455 24391639 PMC3867683

[18] Miller JD van der Most RG Akondy RS Glidewell JT Albott S Masopust D. Human effector and memory CD8+ T cell responses to smallpox and yellow fever vaccines. Immunity. (2008) 28:710–22. 10.1016/j.immuni.2008.02.020 18468462

[19] Attanasio J Wherry EJ. Costimulatory and coinhibitory receptor pathways in infectious disease. Immunity. (2016) 44:1052–68. 10.1016/j.immuni.2016.04.022 27192569 PMC4873956

[20] Bengsch B Seigel B Ruhl M Timm J Kuntz M Blum HE. Coexpression of PD-1, 2B4, CD160 and KLRG1 on exhausted HCV-specific CD8+ T cells is linked to antigen recognition and T cell differentiation. PLoS Pathog. (2010) 6:e1000947. 10.1371/journal.ppat.1000947 20548953 PMC2883597

[21] Hoffmann M Pantazis N Martin GE Hickling S Hurst J Meyerowitz J. Exhaustion of activated CD8 T cells predicts disease progression in primary HIV-1 infection. PLoS Pathog. (2016) 12:e1005661. 10.1371/journal.ppat.1005661 27415828 PMC4945085

[22] Paley MA Kroy DC Odorizzi PM Johnnidis JB Dolfi DV Barnett BE. Progenitor and terminal subsets of CD8+ T cells cooperate to contain chronic viral infection. Science. (2012) 338:1220–5. 10.1126/science.1229620 23197535 PMC3653769

[23] McLane LM Abdel-Hakeem MS Wherry EJ. CD8 T cell exhaustion during chronic viral infection and cancer. Annu Rev Immunol. (2019) 37:457–95. 10.1146/annurev-immunol-041015-055318 30676822

[24] Kasprowicz V Schulze zur Wiesch J Kuntzen T Nolan BE Longworth S Berical A. High level of PD-1 expression on Hepatitis C Virus (HCV)-specific CD8+ and CD4+ T cells during acute HCV infection, irrespective of clinical outcome. J Virol. (2008) 82:3154–60. 10.1128/JVI.02474-07 18160439 PMC2258997

[25] Angelosanto JM Blackburn SD Crawford A Wherry EJ. Progressive loss of memory T cell potential and commitment to exhaustion during chronic viral infection. J Virol. (2012) 86:8161–70. 10.1128/JVI.00889-12 22623779 PMC3421680

[26] Abel A Steeg C Aminkiah F Addai-Mensah O Addo M Gagliani N Differential expression pattern of co-inhibitory molecules on CD4+ T cells in uncomplicated versus complicated malaria. Sci Rep. (2018) 8:4789 10.1038/s41598-018-22659-1 29555909 PMC5859076

[27] Mackroth MS Abel A Steeg C Schulze zur Wiesch J Jacobs T. Acute malaria induces PD1+CTLA4+ effector T cells with cell-extrinsic suppressor function. PLoS Pathog. (2016) 12:e1005909. 10.1371/journal.ppat.1005909 27802341 PMC5089727

[28] Lyke KE Burges R Cissoko Y Sangare L Dao M Diarra I Serum levels of the proinflammatory cytokines interleukin-1 beta (IL-1β), IL-6, IL-8, IL-10, tumor necrosis factor alpha, and IL-12(p70) in Malian children with severe Plasmodium falciparum malaria and matched uncomplicated malaria or healthy controls. Infect Immun. (2004) 72:5630–7. 10.1128/IAI.72.10.5630-5637.2004 15385460 PMC517593

[29] Jacobs T Plate T Gaworski I Fleischer B. CTLA-4-dependent mechanisms prevent T cell induced-liver pathology during the erythrocyte stage of *Plasmodium berghei* malaria. Eur J Immunol. (2004) 34:972–80. 10.1002/eji.200324477 15048707

[30] Costa PAC Leoratti FMS Figueiredo MM Tada MS Pereira DB Junqueira C. Induction of inhibitory receptors on T cells during plasmodium vivax malaria impairs cytokine production. J Infect Dis. (2015) 212:1999–2010. 10.1093/infdis/jiv306 26019284 PMC4655853

[31] Butler NS Moebius J Pewe LL Traore B Doumbo OK Tygrett LT. Therapeutic blockade of PD-L1 and LAG-3 rapidly clears established blood-stage *Plasmodium* infection. Nat Immunol. (2012) 13:188–95. 10.1038/ni.2180 22157630 PMC3262959

[32] Dookie RS Villegas-Mendez A Kroeze H Barrett JR Draper SJ Franke-Fayard BM. Combinatorial Tim-3 and PD-1 activity sustains antigen-specific Th1 cell numbers during blood-stage malaria. Parasite Immunol. (2020). 10.1111/pim.12723. [Epub ahead of print]. 32306409

[33] Fuertes Marraco SA Neubert NJ Verdeil G Speiser DE. Inhibitory receptors beyond T cell exhaustion. Front Immunol. (2015) 6:310. 10.3389/fimmu.2015.00310 26167163 PMC4481276

[34] Jubel JM Barbati ZR Burger C Wirtz DC Schildberg FA. The role of PD-1 in acute and chronic infection. Front Immunol. (2020) 11:487. 10.3389/fimmu.2020.00487 32265932 PMC7105608

[35] Nörz D Fischer N Schultze A Kluge S Mayer-Runge U Aepfelbacher M. Clinical evaluation of a SARS-CoV-2 RT-PCR assay on a fully automated system for rapid on-demand testing in the hospital setting. J Clin Virol. (2020) 128:104390. 10.1016/j.jcv.2020.104390 32388471 PMC7187839

[36] Dunay GA Tóth I Eberhard JM Degen O Tolosa E van Lunzen J. Parallel assessment of Th17 cell frequencies by surface marker co-expression versus *ex vivo* IL-17 production in HIV-1 infection. Cytom Part B Clin Cytom. (2016) 90:486–92. 10.1002/cyto.b.21352 26666875

[37] Callan MFC Tan L Annels N Ogg GS Wilson JDK O'Callaghan CA. Direct visualization of antigen-specific CD8+ T cells during the primary immune response to Epstein-Barr virus *in vivo*. J Exp Med. (1998) 187:1395–402. 10.1084/jem.187.9.1395 9565632 PMC2212279

[38] Thevarajan I Nguyen THO Koutsakos M Druce J Caly L van de Sandt CE. Breadth of concomitant immune responses prior to patient recovery: a case report of non-severe COVID-19. Nat Med. (2020) 26:453–5. 10.1038/s41591-020-0819-2 32284614 PMC7095036

[39] Wittner M Schlicker V Libera J Bockmann JH Horvatits T Seiz O. Comparison of the integrin α4β7 expression pattern of memory T cell subsets in HIV infection and ulcerative colitis. PLoS ONE. (2019) 14:e0220008. 10.1371/journal.pone.0220008 31356607 PMC6663001

[40] Gilfillan S Chan CJ Cella M Haynes NM Rapaport AS Boles KS. DNAM-1 promotes activation of cytotoxic lymphocytes by nonprofessional antigen-presenting cells and tumors. J Exp Med. (2008) 205:2965–73. 10.1084/jem.20081752 19029380 PMC2605240

[41] Cella M Presti R Vermi W Lavender K Turnbull E Ochsenbauer-Jambor C. Loss of DNAM-1 contributes to CD8+ T-cell exhaustion in chronic HIV-1 infection. Eur J Immunol. (2010) 40:949–54. 10.1002/eji.200940234 20201043 PMC3031090

[42] Blackburn SD Shin H Haining WN Zou T Workman CJ Polley A. Coregulation of CD8+ T cell exhaustion by multiple inhibitory receptors during chronic viral infection. Nat Immunol. (2009) 10:29–37. 10.1038/ni.1679 19043418 PMC2605166

[43] Mohammadizad H Shahbazi M Hasanjani Roushan MR Soltanzadeh-Yamchi M Mohammadnia-Afrouzi M. TIM-3 as a marker of exhaustion in CD8 + T cells of active chronic hepatitis B patients. Microb Pathog. (2019) 128:323–8. 10.1016/j.micpath.2019.01.026 30660734

[44] David P Megger DA Kaiser T Werner T Liu J Chen L. The PD-1/PD-L1 pathway affects the expansion and function of cytotoxic CD8+ T cells during an acute retroviral infection. Front Immunol. (2019) 10:54. 10.3389/fimmu.2019.00054 30804928 PMC6370637

[45] Gorman JV Colgan JD. Acute stimulation generates Tim-3-expressing T helper type 1 CD4 T cells that persist *in vivo* and show enhanced effector function. Immunology. (2018) 154:418–33. 10.1111/imm.12890 29315553 PMC6002241

[46] Richter K Agnellini P Oxenius A. On the role of the inhibitory receptor LAG-3 in acute and chronic LCMV infection. Int Immunol. (2010) 22:13–23. 10.1093/intimm/dxp107 19880580

[47] Doe HT Kimura D Miyakoda M Kimura K Akbari M Yui K. Expression of PD-1/LAG-3 and cytokine production by CD4+ T cells during infection with *Plasmodium parasites*. Microbiol Immunol. (2016) 60:121–31. 10.1111/1348-0421.12354 26696540

[48] Adler G Steeg C Pfeffer K Murphy TL Murphy KM Langhorne J. B and T lymphocyte attenuator restricts the protective immune response against experimental malaria. J Immunol. (2011) 187:5310–9. 10.4049/jimmunol.1101456 21998455

[49] Otsuki N Kamimura Y Hashiguchi M Azuma M. Expression and function of the B and T lymphocyte attenuator (BTLA/CD272) on human T cells. Biochem Biophys Res Commun. (2006) 344:1121–7. 10.1016/j.bbrc.2006.03.242 16643847

[50] Sullivan BM Juedes A Szabo SJ Von Herrath M Glimcher LH. Antigen-driven effector CD8 T cell function regulated by T-bet. Proc Natl Acad Sci USA. (2003) 100:15818–23. 10.1073/pnas.2636938100 14673093 PMC307651

[51] Popescu I Pipeling MR Shah PD Orens JB McDyer JF. T-bet:eomes balance, effector function, and proliferation of cytomegalovirus-specific CD8 + T cells during primary infection differentiates the capacity for durable immune control. J Immunol. (2014) 193:5709–22. 10.4049/jimmunol.1401436 25339676 PMC4239205

[52] Intlekofer AM Takemoto N Wherry EJ Longworth SA Northrup JT Palanivel VR. Effector and memory CD8+ T cell fate coupled by T-bet and eomesodermin. Nat Immunol. (2005) 6:1236–44. 10.1038/ni1268 16273099

[53] van Aalderen MC Remmerswaal EBM Verstegen NJM Hombrink P Ten Brinke A Pircher H. Infection history determines the differentiation state of human CD8 + T cells. J Virol. (2015) 89:5110–23. 10.1128/JVI.03478-14 25717102 PMC4403462

[54] Knox JJ Cosma GL Betts MR McLane LM. Characterization of T-bet and Eomes in peripheral human immune cells. Front Immunol. (2014) 5:217. 10.3389/fimmu.2014.00217 24860576 PMC4030168

[55] Buggert M Tauriainen J Yamamoto T Frederiksen J Ivarsson MA Michaëlsson J. T-bet and eomes are differentially linked to the exhausted phenotype of CD8+ T cells in HIV infection. PLoS Pathog. (2014) 10:e1004251. 10.1371/journal.ppat.1004251 25032686 PMC4102564

[56] Ribeiro-dos-Santos P Turnbull EL Monteiro M Legrand A Conrod K Baalwa J. Chronic HIV infection affects the expression of the 2 transcription factors required for CD8 T-cell differentiation into cytolytic effectors. Blood. (2012) 119:4928–38. 10.1182/blood-2011-12-395186 22490682

[57] Schluns KS Kieper WC Jameson SC Lefrançois L. Interleukin-7 mediates the homeostasis of naïve and memory CD8 T cells *in vivo*. Nat Immunol. (2000) 1:426–32. 10.1038/80868 11062503

[58] Tan JT Dudl E LeRoy E Murray R Sprent J Weinberg KI. IL-7 is critical for homeostatic proliferation and survival of naïve T cells. Proc Natl Acad Sci USA. (2001) 98:8732–7. 10.1073/pnas.161126098 11447288 PMC37504

[59] Kaech SM Tan JT Wherry EJ Konieczny BT Surh CD Ahmed R. Selective expression of the interleukin 7 receptor identifies effector CD8 T cells that give rise to long-lived memory cells. Nat Immunol. (2003) 4:1191–8. 10.1038/ni1009 14625547

[60] Boutboul F Puthier D Appay V Pellé O Ait-Mohand H Combadière B. Modulation of interleukin-7 receptor expression characterizes differentiation of CD8 T cells specific for HIV, EBV and CMV. AIDS. (2005) 19:1981–6. 10.1097/01.aids.0000191919.24185.46 16260904

[61] Kasprowicz V Kang YH Lucas M Schulze zur Wiesch J Kuntzen T Fleming V. Hepatitis C virus (HCV) sequence variation induces an HCV-specific T-cell phenotype analogous to spontaneous resolution. J Virol. (2010) 84:1656–63. 10.1128/JVI.01499-09 19906915 PMC2812309

[62] Anderson AC Joller N Kuchroo VK. Lag-3, Tim-3, and TIGIT: co-inhibitory receptors with specialized functions in immune regulation. Immunity. (2016) 44:989–1004. 10.1016/j.immuni.2016.05.001 27192565 PMC4942846

[63] Kaminski LC Riehn M Abel A Steeg C Yar DD Addai-Mensah O. Cytotoxic T cell-derived granzyme B is increased in severe plasmodium falciparum malaria. Front Immunol. (2019) 10:2917. 10.3389/fimmu.2019.02917 31921176 PMC6918797

[64] Niu B Zhou F Su Y Wang L Xu Y Yi Z. Different expression characteristics of LAG3 and PD-1 in sepsis and their synergistic effect on T cell exhaustion: a new strategy for immune checkpoint blockade. Front Immunol. (2019) 10:1888. 10.3389/fimmu.2019.01888 31440257 PMC6693426

[65] Zhang P Lee JS Gartlan KH Schuster IS Comerford I Varelias A. Eomesodermin promotes the development of type 1 regulatory T (TR1) cells. Sci Immunol. (2017) 2:eaah7152. 10.1126/sciimmunol.aah7152 28738016 PMC5714294

[66] Gruarin P Maglie S De Simone M Häringer B Vasco C Ranzani V. Eomesodermin controls a unique differentiation program in human IL-10 and IFN-γ coproducing regulatory T cells. Eur J Immunol. (2019) 49:96–111. 10.1002/eji.201847722 30431161

[67] Kao C Oestreich KJ Paley MA Crawford A Angelosanto JM Ali MAA. Transcription factor T-bet represses expression of the inhibitory receptor PD-1 and sustains virus-specific CD8+ T cell responses during chronic infection. Nat Immunol. (2011) 12:663–71. 10.1038/ni.2046 21623380 PMC3306165

[68] Lozano E Dominguez-Villar M Kuchroo V Hafler DA. The TIGIT/CD226 axis regulates human T cell function. J Immunol. (2012) 188:3869–75. 10.4049/jimmunol.1103627 22427644 PMC3324669

[69] Robilotti EV Babady NE Mead PA Rolling T Perez-Johnston R Bernardes M Determinants of COVID-19 disease severity in patients with cancer. Nat Med. (2020) 1–6. 10.1038/s41591-020-0979-0 32581323 PMC7785283

[70] Huang KJ Su IJ Theron M Wu YC Lai SK Liu CC. An interferon-γ-related cytokine storm in SARS patients. J Med Virol. (2005) 75:185–94. 10.1002/jmv.20255 15602737 PMC7166886

[71] Younan P Iampietro M Nishida A Ramanathan P Santos RI Dutta M. Ebola virus binding to Tim-1 on T lymphocytes induces a cytokine storm. MBio. (2017) 8:e00845–17. 10.1128/mBio.00845-17 28951472 PMC5615193

[72] Teijaro JR Walsh KB Cahalan S Fremgen DM Roberts E Scott F. Endothelial cells are central orchestrators of cytokine amplification during influenza virus infection. Cell. (2011) 146:980–91. 10.1016/j.cell.2011.08.015 21925319 PMC3176439

[73] Zhou Y Fu B Zheng X Wang D Zhao C Qi Y Pathogenic T-cells and inflammatory monocytes incite inflammatory storms in severe COVID-19 patients. Natl Sci Rev. (2020) 2020:nwaa041 10.1093/nsr/nwaa041 PMC7108005 34676125

[74] Ruibal P Oestereich L Ludtke A Becker-Ziaja B Wozniak DM Kerber R. Unique human immune signature of Ebola virus disease in Guinea. Nature. (2016) 533:100–4. 10.1038/nature17949 27147028 PMC4876960

[75] Baitsch L Legat A Barba L Fuertes Marraco SA Rivals JP Baumgaertner P. Extended co-expression of inhibitory receptors by human CD8 T-cells depending on differentiation, antigen-specificity and anatomical localization. PLoS ONE. (2012) 7:e30852. 10.1371/journal.pone.0030852 22347406 PMC3275569

[76] Kroy DC Ciuffreda D Cooperrider JH Tomlinson M Hauck GD Aneja J. Liver environment and HCV replication affect human T-cell phenotype and expression of inhibitory receptors. Gastroenterology. (2014) 146:550–61. 10.1053/j.gastro.2013.10.022 24148617 PMC3946973

