# Supplementary material for: Analysis of Co-inhibitory Receptor Expression in COVID-19 Infection Compared to Acute Plasmodium falciparum Malaria: LAG-3 and TIM-3 Correlate With T Cell Activation and Course of Disease
Source: Front Immunol. 2020 Aug 26;11:1870. doi: 10.3389/fimmu.2020.01870 (PMC7479337; doi:10.3389/fimmu.2020.01870)
Supplement: Supplementary file 1 [file Data_Sheet_1.pdf]

Supplementary Figure 1

Panel 1

Fluorchrome	Antigen	Clone	Provider
BUV 737	CD4	SK3	BD Biosciences
BUV 395	CD38	HB7	BD Biosciences
BV785	TIM-3	F38-2E2	Biolegend
BV711	HLA-DR	L243	Biolegend
BV650	CCR7	G043H7	Biolegend
BV605	TIGIT	A15153G	Biolegend
BV510	CD45RO	UCHL1	Biolegend
BV421	LAG-3	11C3C65	Biolegend
PerCp-Cy5.5	CD8	RPA-T8	Biolegend
FITC	CD27	M-T271	BD Biosciences
PE-Cy7	CD69	FN50	Biolegend
PE-Dazzle	PD1	EH12.2H7	Biolegend
PE	BTLA	J168-540	BD Biosciences
APC-Cy7	L/D		ThermoFisher
	CD14	63D3	Biolegend
	CD19	HIB19	Biolegend
AF700	CD3	UCHT1	Biolegend

Panel 2

Fluorchrome	Antigen	Clone	Provider
BUV 737	CD4	SK3	BD Biosciences
BUV 395	CD226	DX11	BD Biosciences
BV785	CD127	A019D5	Biolegend
BV711	T-bet	4B10	Biolegend
BV650	CCR7	G043H7	Biolegend
BV605	TIGIT	A15153G	Biolegend
BV510	CD45RO	UCHL1	Biolegend
BV421	PD1	EH12.2H7	Biolegend
PerCp-Cy5.5	CD8	RPA-T8	Biolegend
FITC	CD27	M-T271	BD Biosciences
PE-Cy7	LAG-3	3DS223H	eBioscience
PE-Dazzle	Eomes	WD1928	Invitrogen
APC-Cy7	L/D		ThermoFisher
	CD14	63D3	Biolegend
	CD19	HIB19	Biolegend
AF700	CD3	UCHT1	Biolegend

Supplementary Figure 1. Overview of fluorochrome-conjugated antibodies for flow cytometry.

Supplementary Figure 2

Identifier	Panel 1	Panel 2	Mild	Severe	Follow-Up
C1		X			
C2	X	X	X		
C3	X	X			
C4	X			X	X
C5	X	X		X	
C6	X	X	X		
C7	X	X			
C8	X	x	X		
C9		X	X		
C10	X	X		X	
C11	X	X	X		
C12	X	X		X	X
C13	X				
C14	X	X			
C15	X	X	X		
C16	X	X			
C17	X				
C18	X			X	
C19	X				
C20	X	X			X
M1	X	X			
M2	X	X			
M3		X			
M4	X	X			
M5		X			
M6		X			
M7	X	X			
M8	X	X			
M9		X			
M10	X	X			

C1-C20: COVID-19 cohort M1-M10: Malaria cohort

Supplementary Figure 2. Overview of processed samples and panels used for analysis.

Supplementary Figure 3

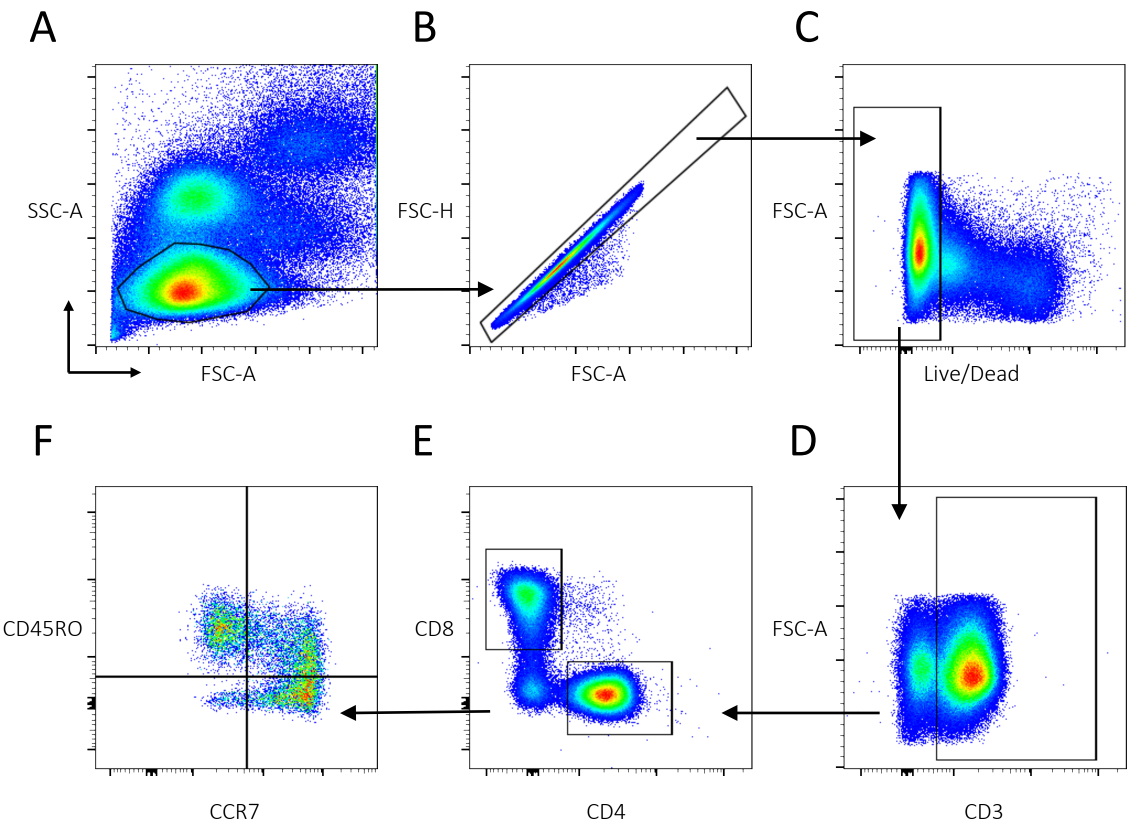

Supplementary Figure 3. Representative dot plots of PBMC showing the gating strategy for T cells. (A) Lymphocytes were first identified by a low forward scatter (FSC) and low side scatter (SSC) gate. **(B)** Doublets were excluded by a forward scatter height (FSC-H) and forward scatter area (FSC-A) gate. **(C)** Only live cells were included. **(D)** Only $CD3^+$ cells were included. **(E)** $CD8^+$ and $CD4^+$ T cells were identified. **(F)** Naïve and memory subsets were identified using CD45RO and CCR7.

Supplementary Figure 4

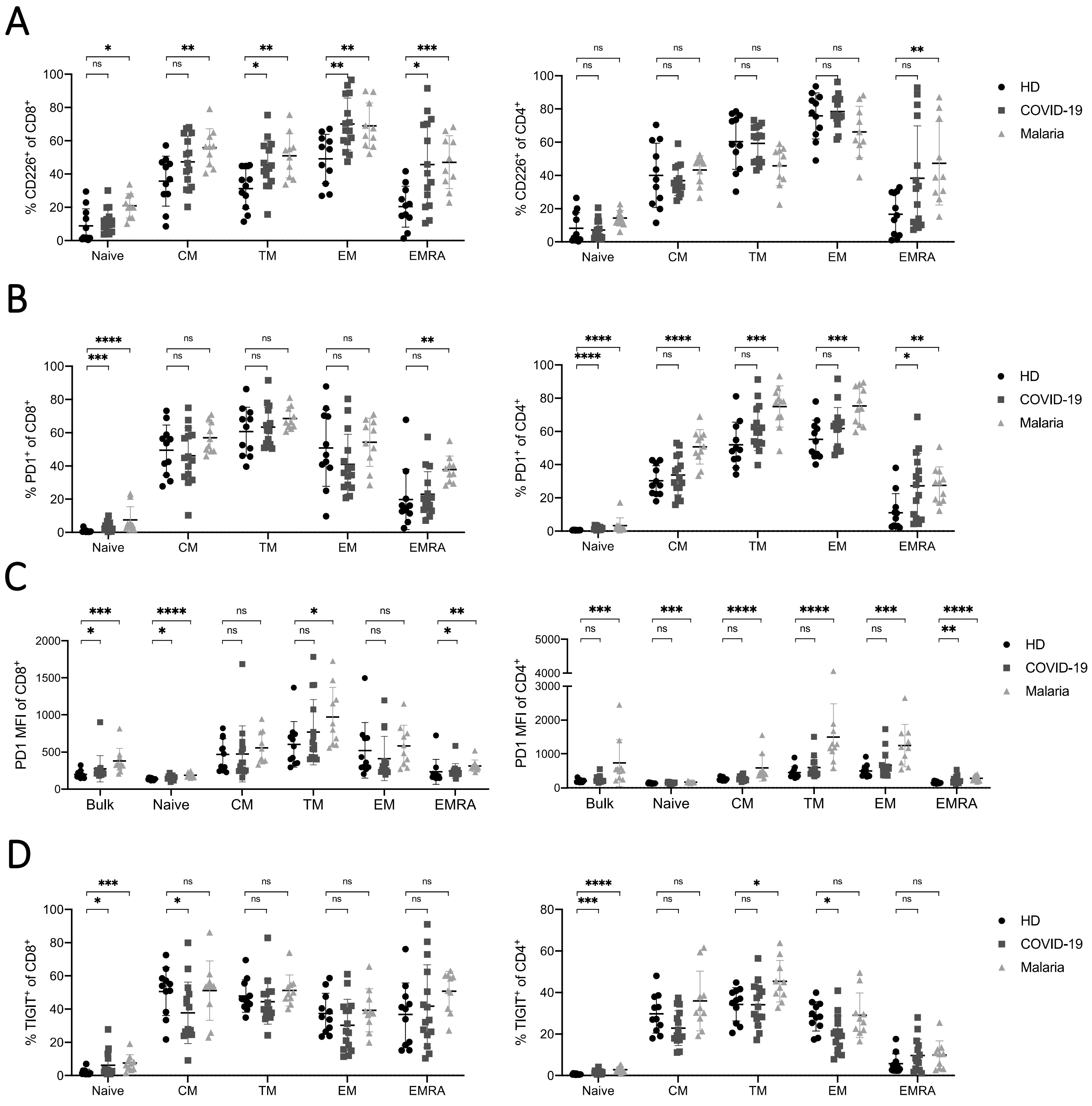

Supplementary Figure 4. Frequency of co-stimulatory and co-inhibitory receptors on naïve and memory subsets. Frequency of CD226 **(A)**, PD1 **(B)**, MFI of PD1 **(C)** and frequency of TIGIT **(D)** on naïve, central memory (CM), transitional memory (TM), effector memory (EM) and terminal effector memory (EMRA) $CD8^+$ and $CD4^+$ T cells in COVID-19 and malaria patients and healthy donors. P values were calculated by Mann-Whitney test. P-values smaller than 0.05 were considered significant, where *, **, *** and **** indicate p-values between 0.01 to 0.05, 0.001 to 0.01, 0.0001 to 0.001 and <0.0001 respectively.

Supplementary Figure 5

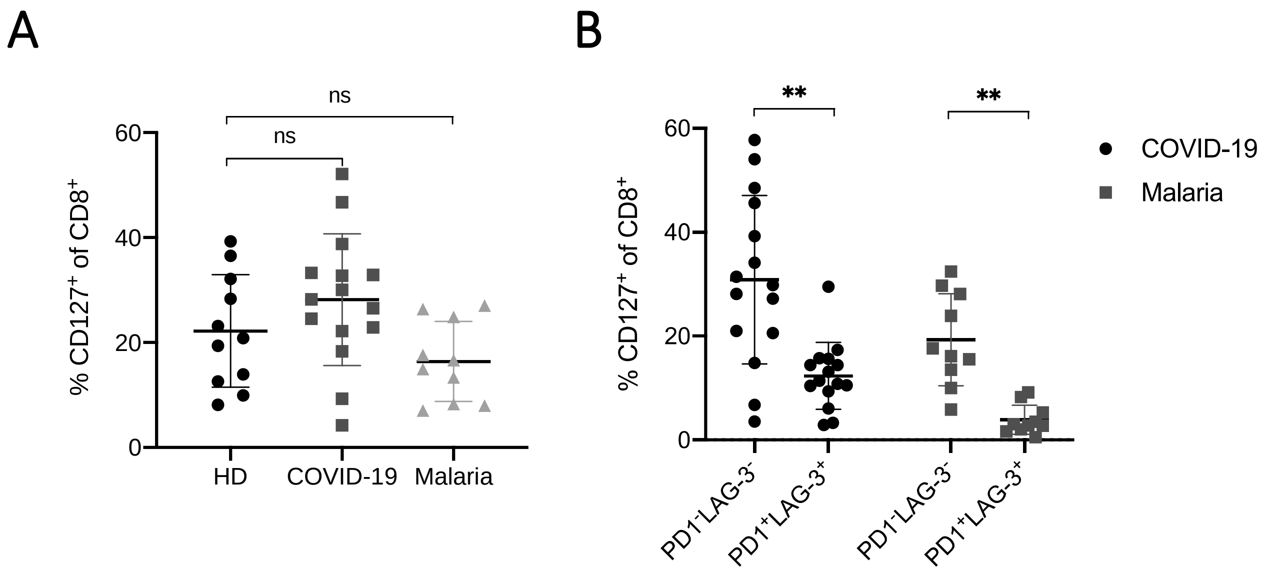

Supplementary Figure 5. Frequency of CD127 on bulk CD8⁺ T cells and CD8⁺ T cells co-expressing inhibitory receptors. (A) Frequency of CD127 on CD8⁺ T cells in COVID-19 and malaria patients as well as healthy donors. **(B)** Frequency of CD127 on PD1⁻LAG-3⁻ and PD1⁺LAG-3⁺ CD8⁺ T cells in COVID-19 and malaria patients. P values were calculated by Wilcoxon test for paired analyses and Mann-Whitney test for unpaired analyses. P-values smaller than 0.05 were considered significant, where *, **, *** and **** indicate p-values between 0.01 to 0.05, 0.001 to 0.01, 0.0001 to 0.001 and <0.0001 respectively.

Supplementary Figure 6

A

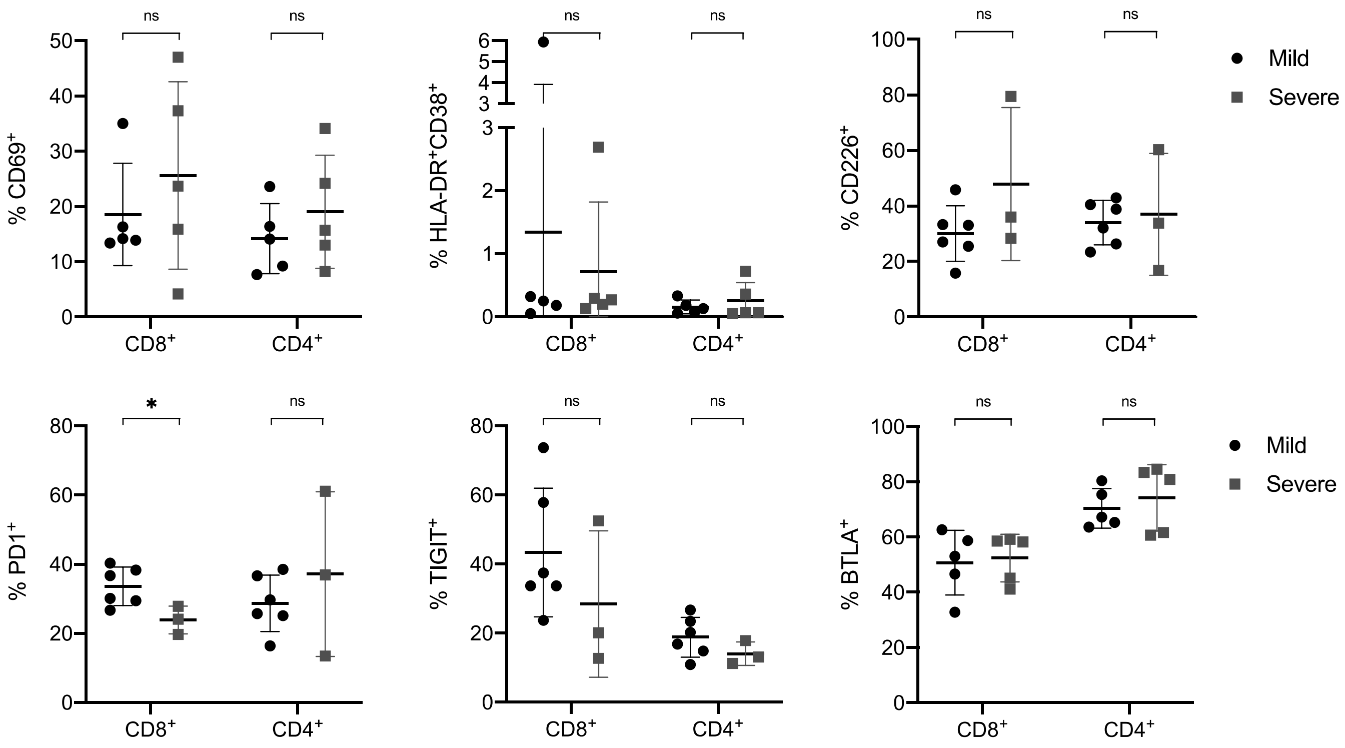

Supplementary Figure 6. Frequency of co-stimulatory and co-inhibitory receptors on CD8⁺ and CD4⁺ T cells in mild and severe COVID-19 patients. (A) Frequencies of CD69⁺, HLA-DR⁺CD38⁺, CD226⁺, PD1⁺, TIGIT⁺ and BTLA⁺ CD8⁺ and CD4⁺ T cells in mild and severe COVID-19 patients. P values were calculated by Mann-Whitney test. P-values smaller than 0.05 were considered significant, where *, **, *** and **** indicate p-values between 0.01 to 0.05, 0.001 to 0.01, 0.0001 to 0.001 and <0.0001 respectively.
